# Analysis of multidimensional impacts of electric vehicles penetration in distribution networks

**DOI:** 10.1038/s41598-024-77662-6

**Published:** 2024-11-13

**Authors:** Rania A. Ibrahim, Ibrahim. M. Gaber, Nahla E. Zakzouk

**Affiliations:** Arab Academy for Science, Technology and Maritime Transport, Alexandria, Egypt

**Keywords:** Electric vehicles, V2G technology, Multidimensional impacts, Challenges, Recommendations, Mitigation, Sustainable development goals (SDGs), Energy grids and networks, Power distribution, Electrical and electronic engineering

## Abstract

Moving towards a cleaner, greener, and more sustainable future, expanding electric vehicles (EVs) adoption is inevitable. However, uncontrolled charging of EVs, especially with their increased penetration among the utility grid, imposes several negative technical impacts, including grid instability and deteriorated power quality in addition to overloading conditions. Hence, smart and coordinated charging is crucial in EV electrification, where Vehicle-to-Grid (V2G) technology is gaining much interest. Owing to its inherited capability of bi-directional power flow, V2G is capable of enhancing grid stability and resilience, load balancing, and congestion alleviation, as well as supporting renewable energy sources (RESs) integration. However, as with most emerging technologies, there are still technical research gaps that need to be addressed. In addition to these technical impacts, other multidisciplinary factors must be investigated to promote EVs adoption and V2G implementation. This paper provides a detailed demonstration of the technical problems associated with EVs penetration in distribution networks along with quantifiable insights into these limitations and the corresponding mitigation schemes. In addition, it discusses V2G benefits for power systems and consumers, as well as explores their technical barriers and research directions to adequately regulate their services and encourage EV’s owners to its embracement. Moreover, other factors, including regulatory, social, economic and environmental ones that affect EV market penetration are being studied and related challenges are analyzed to draw recommendations that aid market growth.

## Introduction

The transportation sector is one of the largest consumers of fossil fuels and a major contributor to greenhouse gases, accounting for more than 20% of the world’s carbon emissions and 30% of the energy consumption worldwide^1,2^. In urban areas, air pollution is most pronounced due to traffic congestion, increased population density and concentration of vehicle emissions. This issue necessitates road transport electrification i.e. replacing internal combustion vehicles with new energy vehicles such as electric vehicles (EV), which appears promising towards achieving urban sustainability. With electrified transportation, a shift to greener transport will take place, reducing reliance on traditional energy sources and realizing energy-saving schemes that are reliable, cost-effective, and environmentally friendly with fewer global gas emissions. This serves well for sustainable development goals (SDGs), notably sdg3: Good Health and Well-being and sdg7: Affordable and clean Energy^3^.

As they achieve market acceptability, the demand for EVs is anticipated to increase significantly especially in smart cities, along with shared mobility and public transport as a replacement for the traditional internal combustion engine (ICE) vehicles^4^. The global EV fleet is predicted to reach 230 million vehicles in 2030, and 58% of vehicles are anticipated to be EVs in 2040^5^. According to^1^, China is the frontrunner, accounting for 60% of global electric car sales in 2022 and is reportedly aiming to ban fossil fuel-powered vehicle sales in the future^6^. Despite all these anticipated projections, large-scale uptake of EVs is limited by several challenges related to the shorter EV driving range compared to that of gasoline-powered vehicles^7^. Hence, to allow large scale EV adoption, adequate and widely spread charging infrastructure is mandatory.

**Fig. 1:**
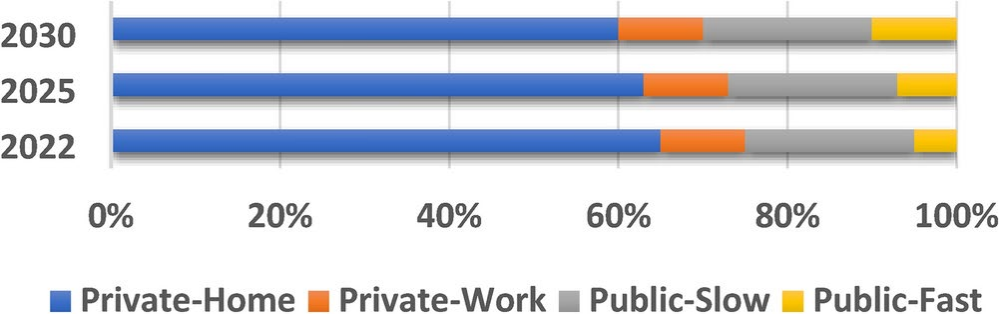
Electricity delivered to electric light-duty vehicles by charger type, 2022–2030^1^.

However, implementing charging infrastructure on a large-scale is competitively costly and associated with several technological challenges, in addition to its noticeable impact on the power grid. With the accelerated adoption of EVs worldwide, several simultaneous charging operations are needed, which affect the utility grid, especially when large amounts of electric energy are required in a short period. Figure 1 depicts the progression of electrical power supplied to light-duty EVs by employing different charger types between 2022 and 2030. Notably, the capacity expansion of fast public chargers profoundly outperforms that of public slow chargers by a factor of 13 and surpasses that of private chargers by 20 times^1^. However, placing fast chargers can induce stresses on the existing electrical infrastructure, leading to negative consequences for the power grid which may necessitate costly utility upgrades. Moreover, uncontrolled charging activities pose significant challenges to local grid stability and may cause voltage variations and imbalances as well as transformer and transmission network overloads in addition to large network losses and harmonics^8,9^. To address these negative technical impacts imposed on the utility grid by the vast penetration of EVs charging stations, coordinated charging approaches along with vehicle-to-grid technology (V2G) are promising^10^.

V2G technology allows bidirectional flow of power between EVs and the power grid via advanced communication systems and smart charging framework. Hence, EV owners have the flexibility to either draw electricity from the grid or inject surplus power back into it during periods of high demand, thus maximizing their financial benefits^11^. Moreover, V2G technology’s potential extends beyond being just an energy storage system for EVs since it can have significant positive impacts on the entire grid i.e. it can improve grid stability, reliability, and flexibility, act as a spinning reserve, emergency backup power and storage for RESs excess and achieve load balancing and congestion management functions^12^. Thus, V2G technology has promising potential for reshaping the EV charging landscape, making it more efficient, cost-effective, and environmentally friendly. However, like most emerging technologies, there are still technical barriers facing V2G such as the need for EV charging scheduling, coordination and infrastructure planning, EV battery handling and disposal, V2G communication systems and cybersecurity^13–16^. These obstacles require further research for a more sustainable and expandable future and to take prominent measures that would accelerate the adoption of this technology and increase its market share.

However, not only technical impacts would arise from EV penetration and V2G technology adoption. Other factors may include policies and regulations, and social acceptance, in addition to economic and environmental aspects which are still under study; but not broadly investigated^2,6,17^. Thus, studies need to be directed towards these aspects to close potential knowledge gaps which in turn aids in promoting V2G technology among consumers and governments to hasten its market growth.

Table 1 compares what the most recent publications on the impacts of EV penetration have included in their study, including direct effects on the grid, influences, and barriers at various levels and whether any recommendations were drawn. It is clear that^18–20^, are concerned with the effect of EV penetration into the distribution network, associated problems, and respective solutions along with potential technical challenges without involving any other factors. In contrast, studies in^14,15,21,22^ did not discuss the problems reflected in the grid resulting from EV penetration although they studied more than one influencing factor. In^21^, the authors studied technological and economic factors, whereas in^14^, technical, economic, and environmental factors were covered. On the contrary^15^, studied all the factors except regulatory ones, whereas^22^ involved them all. Although^6,9,12,16,17,23^ discussed associated problems and solutions when integrating EVs to grid along with studying more than one influencing factor, not all factors from different orientations were covered. Finally, no recommendations or suggestions are provided by^9,12,24^ to promote EV adoption. Comparatively, the work in this paper outweighs all the latter, in the detailed demonstration on the problems associated with EV integration to utility while sorting quantified insights and presenting viable solutions and mitigation techniques as well as pinning out benefits of this integration. Moreover, critical factors from all levels (technological, social, economic, regulatory, and environmental) are studied and key findings are highlighted. Then, for each factor, challenges and barriers are listed and related to findings to drive recommendations and measures that help accelerate EV adoption and increase V2G acceptance among society and governments.

**Table 1:** Most recent studies addressing impacts of EV penetration onto power grids.

References	Effect on distribution network			Impacts (influences and challenges)					Recommendations and measures
	Quantified insights	Problems/solutions	Benefits	Technical	Regulatory/political	Economic	Social	Environmental	
^6^	─	√	√	√	─	√	─	√	√
^9^	√	√	─	√	√	√	─	─	─
^18^	√	√	√	√	─	─	─	─	√
^23^	─	√	√	√	─	√	─	─	√
^17^	√	√	√	√	√	√	─	─	√
^12^	√	√	√	√	√	─	─	─	─
^21^	─	─	√	√	─	√	─	─	√
^22^	─	─	─	√	√	√	√	√	√
^15^	─	─	√	√	─	√	√	√	─
^16^	√	√	√	√	─	√	√	─	√
^14^	─	─	─	√	─	√	─	√	√
^19^	─	√	√	√	─	─	─	─	√
^20^	√	√	─	√	─	─	─	─	√
Proposed	√	√	√	√	√	√	√	√	√

Hence, the significance of this paper resides in the deep study and thorough analysis carried out on the influencing factors of EV penetration in a grid, covering the following points:

- Current EV charging technologies, infrastructure and operation modes for integration into the grid.
- A strong foundation for technical impacts, including the following:
  - Sorted insights and quantified analysis of the negative impacts of EV penetration into the grid along with the respective mitigation schemes.
  - Investigation of V2G technology and its related economics benefits and auxiliary services offered to the grid.
  - Technical barriers and research gaps facing this technology and decelerating its market growth.
  - Suggested research directions to explore advancements and close knowledge gaps related to this technology
- Going beyond the technical level and studying other factors related to political, economic, social and environmental levels along with investigating respective research challenges.
- By relating key findings and challenges for each aspect, recommendations are drawn, and measures are suggested to be taken by governments, decision makers, manufacturers, stakeholders, consumers, etc., to help remove obstacles and encourage the wider use of EVs.
- Compared with other studies in the literature, the proposed study outperforms others in covering multidimensional impacts related to EV integration in the grid along with establishing quantified insights, highlighting key findings, demonstrating research challenges and finally connecting all the latter to provide tangible conclusions to promote V2G adoption and market growth.

## EV charging infrastructure

EV charging is the process of restoring the energy in EV battery by connecting the EV to a charger or a charging station. There are several EV charging techniques, charger types and charging modes as follows:

### EV charging technologies

**Fig. 2:**
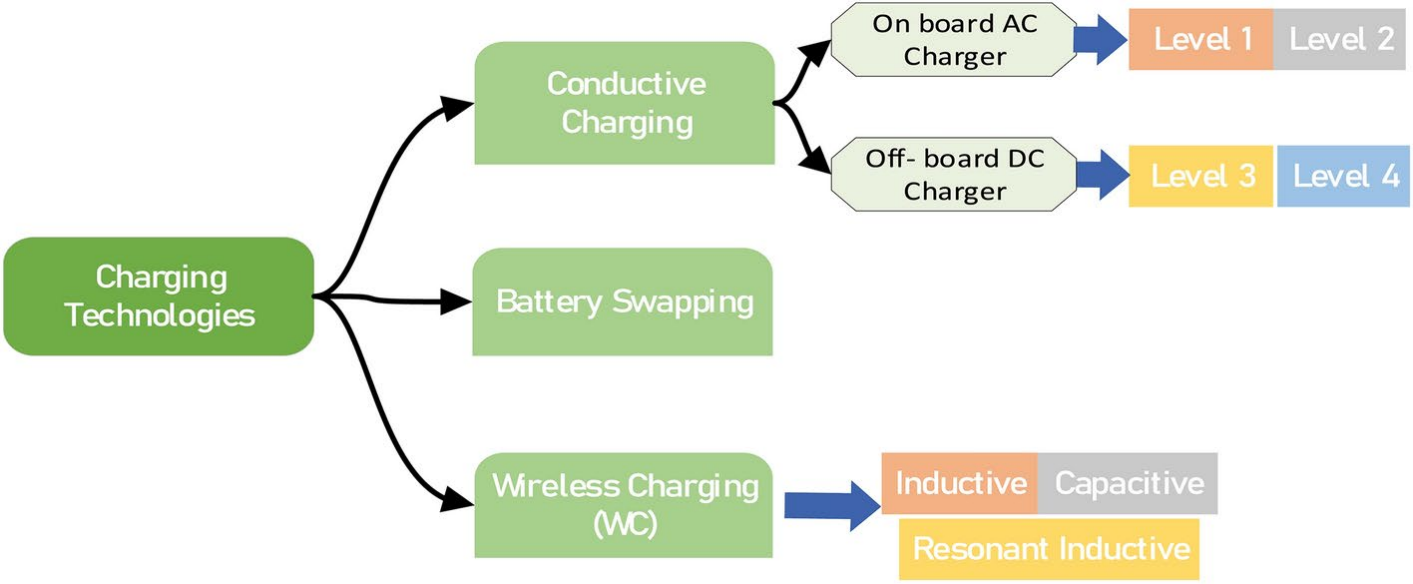
EV charging technologies.

EV charging can be classified into three main technologies: conductive charging, wireless (i.e., contactless) charging and battery swapping as shown in Fig. 2^16,18,19,25^. Conductivity charging is related to the process of charging an EV via direct physical contact (such as a wire) between the power source and the battery. This technology includes on-board and off-board chargers. The former allows the EV to be charged by simply plugging an on-board charger installed on the vehicle itself in any available electrical outlet, whereas off-board chargers are not integrated into the electric vehicle and are typically found in fast-charging stations, business parking lots, and roadways. Although off-board chargers feature greater power transfer capabilities than do on-board chargers, i.e., provide EVs with a higher power charge at a shorter charging duration, they are not available in all places and are more expensive and complex^18,25^. Unlike conductive charging, wireless charging allows an EV to automatically charge without the need for a physical connection between the EV battery and the power source since power transmission is performed via an electromagnetic field^19^. Although this contactless technology can reduce the risk of electric shocks and related damage since power is transmitted through the air gap, it has relatively low charging efficiency because of the existence of a large air gap, noncompliance of the windings and large distances between the charger (transmitter) and the receiver installed on the vehicle. Additionally, the high voltage and high power necessary for EV charging bring additional challenges regarding the cost of wireless charging systems^16^. Battery swapping technology is considered a fast way to fully charge the EV battery where the discharged batteries are replaced by newly charged batteries at a battery swapping station. This reduces the charging time for the EV owner, and the operation and overall efficiency of the power grid can be enhanced by optimal charge and discharge management of batteries at the battery swapping station^19^. Nevertheless, battery mismatch is a challenging issue since the detached battery should have the same characteristics as the newly charged battery that will replace it for efficient performance. Moreover, this technology infrastructure is more complex and expensive than others, which puts more cost on the battery owner when dealing with battery swapping stations^16^.

Despite the merits of wireless and battery swapping technologies, they are still under development to mitigate their limitations. Comparatively, conductive charging is the simplest and currently the most common charging method since it features high charging efficiency at the lowest realization complexity and cost for EV owners, as shown in Table 2^16,26^.

**Table 2:** Comparison of different EV technologies.

Feature	Technique		
	Conductive charging	Wireless charging	Battery swapping
Charging efficiency	High	Low	High
Charging duration	High	High	Low
Infrastructure required	Low	High	Very High
Cost on EV owner	Low	High	High

### EV conductive charger types

As mentioned earlier, two conductivity-based charging strategies exist: (a) an on-board charger and (b) an off-board charger. The former is suitable for AC slow chargers since it features Level 1 and Level 2 charging schemes, while the latter features Level 3 and Level 4 which are convenient for AC and DC fast chargers^27,28^. Thus, conductive charger types can be further subdivided into Level 1, Level 2, Level 3, and Level 4 types on the basis of the charging level as shown in Fig. 2.

The abovementioned charging levels are specified in IEC 61851-1 and SAE J1772^29,30^. They are used to categorize the rated power, voltage, and current of the charging system according to the input current nature (AC or DC) and the power level, which is further reflected in the charging time, as deduced from Table 3. Conductive-based EV chargers are classified into four types: Level 1 chargers, Level 2 chargers, DC fast chargers (Level 3) and ultrafast chargers. Level 1 chargers are typically used at home or workplaces, and they feature low charging capacity by using a standard AC power outlet, whereas Level 2 chargers offer higher charging capacity as well as relatively faster charging capability. These chargers require dedicated charging stations, which can typically be found at public stations such as parking lots or shopping malls, where EVs are parked for an extended period of time. The level 3 DC fast charger, on the other hand, has a much higher charging capacity than the level 1 and 2 types do and can charge an EV considerably faster. Thus, they are used for commercial charging stations and are ideal for long-distance travel or when quick charging is essential. Compared with other levels, level 4 ultrafast chargers offer the lowest charging time, yet at the price of higher installation and capital cost investments^24^. Table 4 shows a schematic of charging ports and connectors manufactured according to different standards. According to the Electric Power Research Institute (EPRI), most EV owners are expected to charge at home at night, so Level 1 and 2 chargers are the main options^31^.

**Table 3:** Comparison between conductive-based charger types.

	Level 1	Level 2	Level 3	Level 4
			
Voltage	120 V AC, 1-phase 250 V AC, 1-phase 480 V AC, 3-phase	208 V- 240 V AC, 1-phase 250 V AC, 1-phase 480 V AC, 3-phase	208/240VAC & 300-800Vdc,	1000 VDC and more
Amps	12A-16A 32 A, 3-phase	12 A-80 A	Up to 500A	More than 400A
Charging mode	AC	AC	AC	DC
Charging time	8–12 h	4–6 h	15–30 min	5 min
Charger	Onboard	Onboard	Offboard	Offboard
Charger type	Slow charging	Moderate charging	Fast charging	Ultrafast charging
Application	Home installation	Public and residential installation	Commercial and public installation	Commercial

**Table 4:** Schematic of charging ports and connectors manufactured according to different standards.

	USA	JAPAN	EU	CHINA	All markets except EU
DC	CCS-1	CHAdeMO	CCS-2	GB/T	Tesla
				
AC	SAE J1772 Leve 1, Level 2 Single Phase	SAE J1772 Leve 1, Level 2 Single Phase	IEC 62,196–2 Level 1,2 Single/Three Phase	IEC 62,196 Level 1,2 Single/Three Phase	
			

### EV charging modes

The International Electrotechnical Commission (IEC) specifies 4 charging modes (IEC-62196 and 61851) for AC and DC charging systems, as shown in Table 5^32^. Mode 1 charging directly connects the electric vehicle to the normal power socket where the charging action is slow and can be used for charging electric scooters and bikes and may be unsafe for use. Owing to the increased power capabilities, Mode 2 demands special safety requirements between the electrical network and the EV. Typical usages for Mode 2 are residential use (up to 16 A) and industrial use (up to 32A). Mode 3, on the other hand, requires that the vehicle must be charged through a permanently connected power supply to the electrical network. The use of wall boxes, commercial charging points and all automatic charging systems in alternating current is a common practice where the car is charged in public places for both slow charging and fast charging. Finally, Mode 4 offers fast charging action through DC charging but at a higher cost.

**Table 5:** EV charging modes.

Charging modes	Power	Charging type	Connection to AC mains	Charging	Notes
Mode 1	AC single and three-phase Up to (3.8–7.6 Kw)	Slow	Passive	Standard household socket (breaker in cable)	Not applicable in some countries manufacturers no longer incorporate this type due to live wire
Mode 2	AC single and three-phase Up to (7.3–15.6 kW)	Slow	Semi-active	Special cable provided with the EV (breaker in cable & pilot function)	Requires control box between vehicle and electrical outlet Prohibited public charging in some countries
Mode 3	AC Single and three-phase Up to (60–120 kW)	Slow or fast	Active	Dedicated charging station/mounted wall box Cable socket provides control and protection	Higher level of safety compared to Mode 2
Mode 4	DC (more than 120 kW)	Fast	Active	Dedicated charging station Special external charger	Not recommended for daily use as it might damage the battery

## EV penetration in a utility grid with V2G technology

A Lack of EV charging stations may impede EV development. Additionally, EV range anxiety (i.e. fear that an electric vehicle will not have enough battery charge to reach its destination) is aggravated by insufficient charging stations and lengthy charging times. To address this issue, the installation of EV charging stations must expand substantially, yet a new demand for electrical utility remains. Compared with overnight slow charging, fast and ultrafast chargers have some different characteristics and present greater implications on the grid, such as more voltage fluctuations, phase imbalances and voltage transients. Additionally, fast charging imposes more harmonics in the line current and harmonic contamination with higher-order harmonics because of the nonlinear behaviour of EV chargers. Moreover, additional impacts can affect distribution system component capacities, power loss, and grid stability^23^. Hence, for most daily charging events, the energy requirement may be met by overnight slow charging. In addition, slow charging features a longer charging duration and a larger distribution area, which facilitates easier planning and control by distribution system operators. By employing bidirectional battery chargers, it is possible to deliver energy in the reverse direction, i.e., from the EV batteries to the power grid, which is known as vehicle-to-grid (V2G) mode^33,34^. This feature facilitates communication between power generation and end-users, thus managing the flow of electricity to meet the fluctuating power demand of end-users. Additionally, this mode can also be utilized to support extensive renewable energy integration and to stabilize the power system^35^. With this developed technology, along with coordinated smart charging techniques, grid stability, resilience and flexibility are enhanced, and the functions of voltage and frequency regulation, load balancing and congestion management can be realized^36^. In addition, EV owners can achieve incentives by selling power to the grid, in addition to providing environmental and economic benefits. Nevertheless, some challenges and regulations should be addressed to encourage the expansion of this technology.

### System components

**Fig. 3:**
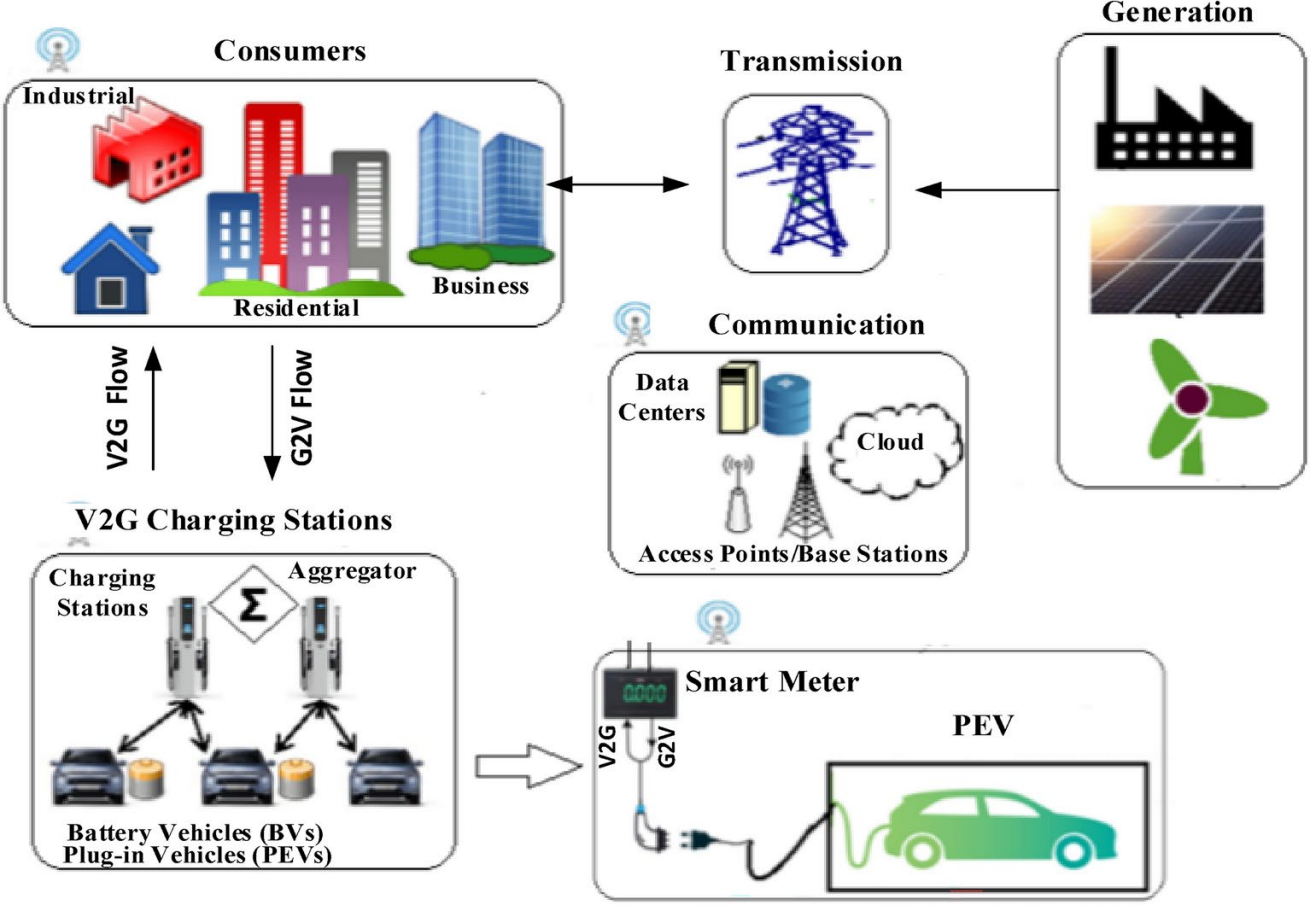
V2G system components and power flow.

Various EVs exist, such as fuel-cell cars, battery-electric cars, and plug-in hybrids. With plug-in hybrid technology, EVs can be operated conventionally or in electric mode. When V2G technology is applied, plug-in EVs (PEVs) require a network connection for power flow, control centers for regulating the charging and discharging processes, communication and a logical interface with the grid operator for signal sending and receiving, as well as an onboard instrumentation system for monitoring. Figure 3 illustrates V2G system components and how EVs and the grid interact. Generators generate electricity that is fed to consumers where EV charging stations can exist. With V2G technology, electricity can be returned to the grid. Grid operators at control centers can transmit control signals via many different methods, the simplest of which are over-the-air, wireless, direct internet links, or power line carriers. Signals can be sent directly to vehicles or received by fleet operators’ offices that manage individual vehicles through distributed power aggregators^36^.

### Power flow

With topologies permitting bi-directional power flow, i.e. grid-to-vehicle (G2V) and vehicle-to-grid (V2G) modes, the two challenges of faster charging and providing ancillary services to the grid can be tackled. With such topologies, two operating modes are included as shown in Fig. 3 and explained below^11^.

a. Grid to Vehicle (G2V)

Typically, EV batteries function as a load when charging in grid-to-vehicle (G2V) mode from the electrical grid. The one-way G2V charging method increases the demand on the electrical system, leading to a potential risk of overloading and overheating power lines as well as distribution transformers^12^. Thus, the V2G added feature is developed not only to reduce the EV charging effects on grid but also to offer grid assistance in frequency and voltage regulation as well as load balancing.

b. Vehicle to Grid (V2G)

V2G technology with bi-directional charging stations, allows owners to charge their vehicles from grid and parked EVs can transform into power providers, because their energy storage systems permit two-way power flow^37^. Thus, EVs can act as portable distributed energy storage systems where during periods of low demand, the onboard battery is connected to the nearby power grid via proper communication equipment, enabling the car batteries to be fully charged and when the vehicle is not in use. On the other hand, the power is provided from EVs to the grid in response to peak load demands. This technology is evolving steadily, especially on the power electronic level of design. Numerous companies are striving to utilize EV batteries as a portable energy source, capable of handling electrical overloads or supplying buildings with power during periods when limited power generation is taking place^38,39^. Other bi-directional concepts, including home-to-vehicle (H2V) and vehicle-for-grid (V4G) systems exist. In addition, improved vehicle-to-home (V2H) mode was presented in^40,41^.

### EV charging influence

**Fig. 4:**
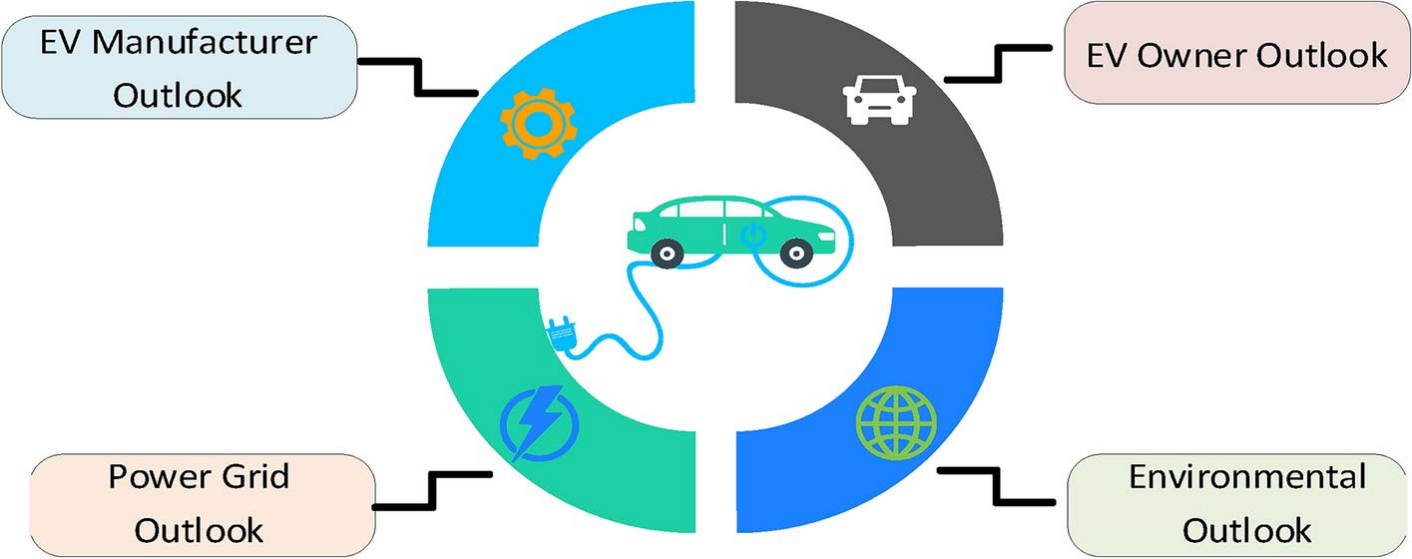
Perspectives of the influence of EV charging.

Investigating the influence of EV charging involves analysing various aspects, such as the level of EV penetration, charging approaches, battery attributes, charging locations and durations, battery state-of-charge, EV fleet charging habits, pricing structures, and demand response methods. According to^42^, the influences of EV charging can be categorized into four main areas, as illustrated in Fig. 4. EV owner perspective, EV manufacturers’ outlook, environmental aspects, and electrical grid impacts. From the standpoint of PEV owners, uncoordinated charging may lead to high charging expenses due to the correlation between PEV arrival times and peak power grid demand, which leads to increased electricity costs. Nevertheless, this drawback can be offset by advantages such as extended battery life and the guarantee of a fully charged vehicle at the time of departure. On the other hand, PEV manufacturers are primarily concerned with battery degradation, as they typically offer warranties for normal battery use under standard conditions. Smart charging, however, can accelerate battery degradation since it reduces the negative effects of charging time and state of charge (SOC) on battery degradation. As a result, PEV manufacturers tend to favour uncoordinated charging strategies. From an environmental standpoint, the use of renewable energy sources (RESs) for charging EVs enhances their overall environmental benefits but at the cost of more RES installations as well as energy storage systems, thus increasing the preference for coordinated smart charging. Finally, from the power grid perspective, uncoordinated charging may impact electricity generation, transmission, and distribution networks^17^. From an electricity generation standpoint, uncoordinated charging may have significant consequences, including capacity expansion issues and rising energy prices. For the transmission network, the influx of EVs may lead to network congestion and raise concerns about overall system reliability. On the low-voltage distribution network level, greater effects may be exhibited, such as overloads of lines and feeders, increased power losses, and power quality concerns. In contrast, V2G introduces several benefits to the grid, such as voltage and frequency regulation and load shaping.

Given that this work focuses primarily on examining the different impacts of EV in the utility distribution network, related research gaps and recommendations, the preceding section will predominantly concentrate on the technical impacts (problems imposed on the grid and their mitigation techniques, V2G benefits followed by technical challenges and recommendations drawn). Other impacts and challenges related to different aspects (regulatory, environmental, economic and social) are discussed in the fifth section.

## Technical impacts of the integration of EVs into grid, related challenges and recommendations

Different technical impacts resulting from EV integration into the grid are thoroughly studied including problems and corresponding mitigation techniques as well as perspective benefits. Furthermore, related research challenges and potential measures are presented.

### Technical problems of EV integration to grid and mitigation strategies

**Fig. 5:**
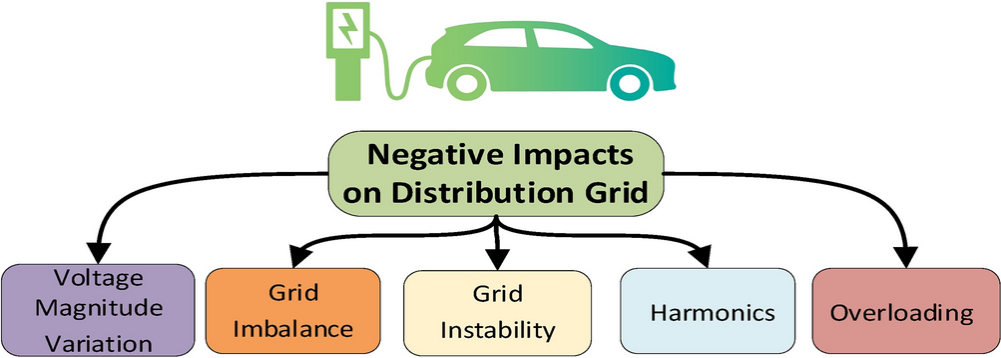
Negative technical impacts of EVs on the distribution electrical grid.

Utility companies encounter notable challenges because of the extensive electric vehicle integration on the distribution network, which disrupts the stability of the grid. The negative consequences stem from voltage level variations and imbalance, grid instability, excessive harmonics introduction and alteration of the load profile and equipment overloading, as indicated in Fig. 5. The overabundance of EVs can lead to serious power quality issues, increased peak loads and challenges in terms of power regulation. Considerable research has been conducted to evaluate the consequences of severe electric vehicle charging, predominantly on the distribution network, since the most severe impacts can be exhibited at this level. Moreover, numerous hardware solutions and software techniques to mitigate these negative impacts have been introduced in the literature.

#### Voltage magnitude variation

**Fig. 6:**
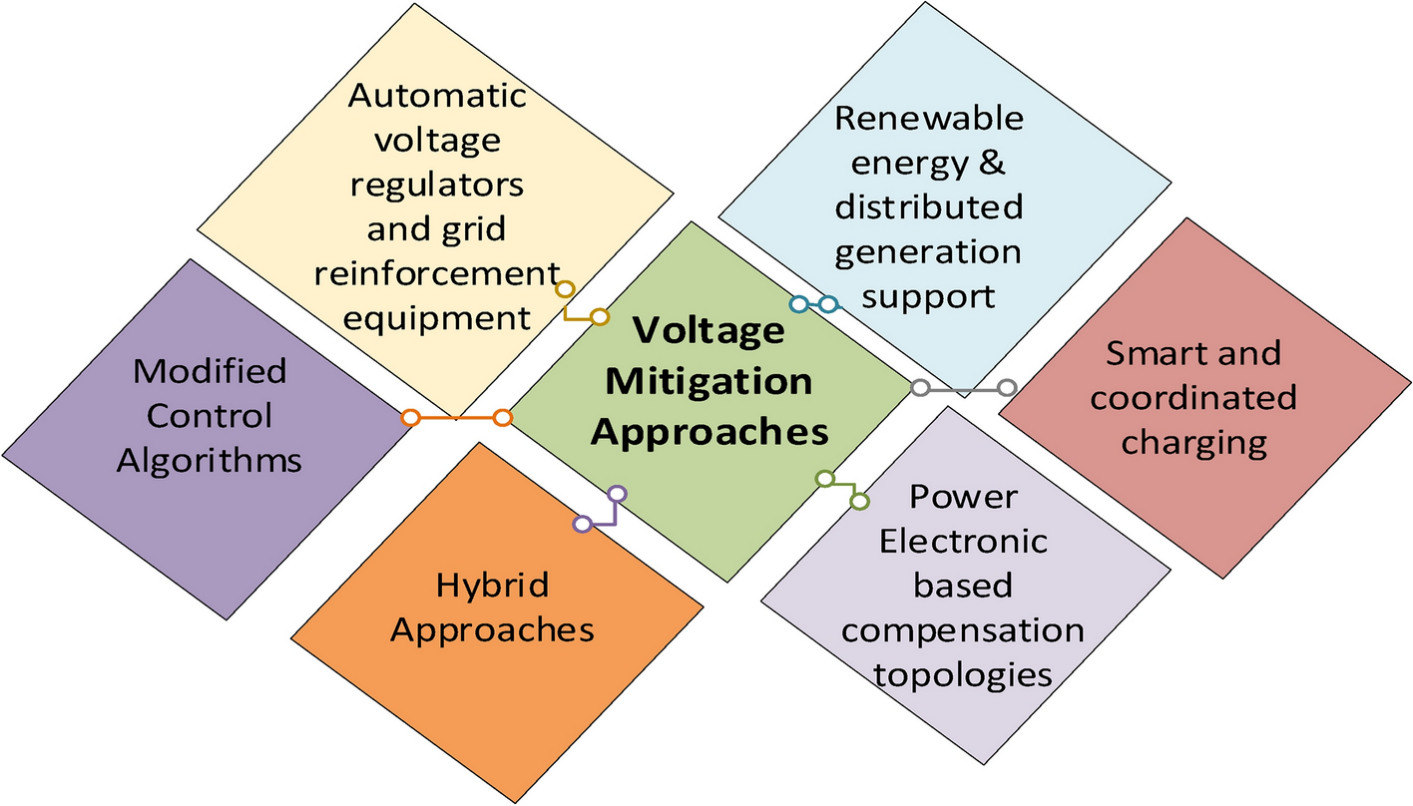
Mitigation techniques for voltage magnitude variations resulting from excessive EV penetration.

Voltage magnitude variation is characterized as a increase or decrease in the root mean square of the instantaneous voltage values, across a specified time period and bandwidth. These variations are specified values assigned by PQ standards such as IEEE 1159 and IEC 61000-2-4. From the utility point of view, an EV can be viewed as a load with a concentration of charging points which leads to dips when many EVs are connected to the grid. Hence, heavy traffic and vehicle colliding with the utility pole tending the wire to touch, are all considered causes of voltage sags resulting from EV equipment typically near the point of use. Nearly all facilities experience voltage sags on an annual basis, ranging from minor fluctuations that wear out and deteriorate equipment to major fluctuations that force equipment and grid operation to become offline^43,44^. This can elevate the cost of line process restoration^45^. Several solutions to voltage level variation issues have been introduced in literature. This can be categorized as depicted in Fig. 6.

Voltage magnitude mitigation techniques in power systems are broadly categorized into centralized and decentralized approaches. Centralized control strategies have enhanced control capabilities as they involve a single controller making decisions for the entire grid. In all cases, centralized control, as shown in Table 6, provides the best voltage variation reduction and feeder voltages maintainability at nominal values with − 4% to + 2% tolerance (minimum value of 0.96 p.u and maximum value of 1.02 p.u). Thus, centralized approaches have improved voltage control capabilities and better mitigation of voltage instability. However, this comes at the cost of less reliability since in case of centralized controller failure, whole system control will fail. Moreover, it requires strong communication infrastructure and can be computationally expensive if implemented on large scale systems^54^. Decentralized approaches, on the other hand, divide the computational burden on multiple controllers, making them more reliable and suitable for large-scale integration^54^, yet with less voltage variations’ reduction capabilities in some cases. Thus, integrating centralized with decentralized methods can be a promising solution^57^.

**Table 6:** Voltages variations reduction approaches implemented in literature.

Ref	System	Control	Results			Mitigation technique
^46,47^	IEEE13 system	Centralized	Voltage p.u, (At node) = 0.96–1.02 kW losses reduction % = 2.65 Peak Demand (kW) reduction % = 4.2%			Droop control-based technique for VAR control using on-load tap changers/AVR and capacitor banks
	Distribution grids with DG, EV battery, and converters	Centralized	Voltage Limits	Before	After	Energy management of EVs bidirectional chargers for voltage support and active power allocation based on state-of-charge (Soc) and battery capacities
			Lower limit	0.94 p.u	0.98 p.u	
			Upper limit	1.06 p.u	1.02 p.u	
^48^	Grid, load with two converter stages	Centralized	Voltage sag improvement from 0.85 to 0.96 p.u			Utilizing intelligent bidirectional power flow charger for voltage sag compensation and active and reactive power support
^49^	20 kV Pujon Feeder system in Malang, Indonesia	Centralized	ΔV% reduction = 7.29%			Optimal PEV charging using Binary Particle Swarm Optimization (BPSO)
^50^	IEEE 14 system	Decentralized	Voltage between 0.9 and 1.1 p.u			Optimal control of P and Q injection from battery storage system at selected buses
^51^	Multiple parallel V2G inverters in Grid-Connected and Stand-Alone Mode	Centralized	ΔV% reduction = 20% (from 0.77 to 0.98 p.u.)			Coordinated virtual impedance control for charging EVs and reducing voltage variations
^52^	IEEE 13 system	Decentralized	Maintain voltage at 0.96 p.u. voltage			Valley-filling charging with regulated nodal voltage magnitudes
^53^	IEEE 37 system	Decentralized	feeder voltage at maximum demand = 0.93 p.u			Integrating EVs using two control levels and negotiation scenario
			feeder voltage after charging point = 0.94 p.u			
			feeder voltage after negotiation scenario = 0.952 p.u			
^54^	IEEE 123 system s	Decentralized	Voltage at 0.945 p.u			Charging stations voltage mitigation by optimal scheduling while meeting users changing needs by a rolling optimization-based approach
^55^	IEEE 37 system	Decentralized	Voltage within 0.95—1.06 p.u			On-board unit with communication between EVs adapting to network voltage
^56^	IEEE European system	Decentralized	without control		0.9–1.03 p.u	Model predictive control to minimize voltage deviation using VAR compensation
			with control		1.01–1.03 p.u	

Optimization and intelligent strategies are applicable for both centralized and decentralized systems for real-time voltage regulation^48–50,54^. Additionally, renewable energy-based voltage mitigation techniques can effectively provide voltage support which is essentially required to accommodate the growing demand on EVs by providing cheaper production and operating costs. However, the intermittent nature of RES can cause instability due to fluctuating generation-load balance, often resulting in power flow reversal and other power quality issues^50,58^. To mitigate these challenges, large-scale BESS are often employed for active and reactive power compensation and thus minimizing the risk of voltage collapse^47^. Furthermore, droop control combined with reactive power support have shown significant potential in reducing voltage variations^46^ as well as applying coordinated virtual impedance control^51^, model predictive control^56^and Valley-filling charging^52^.

#### Voltage imbalance

**Fig. 7:**
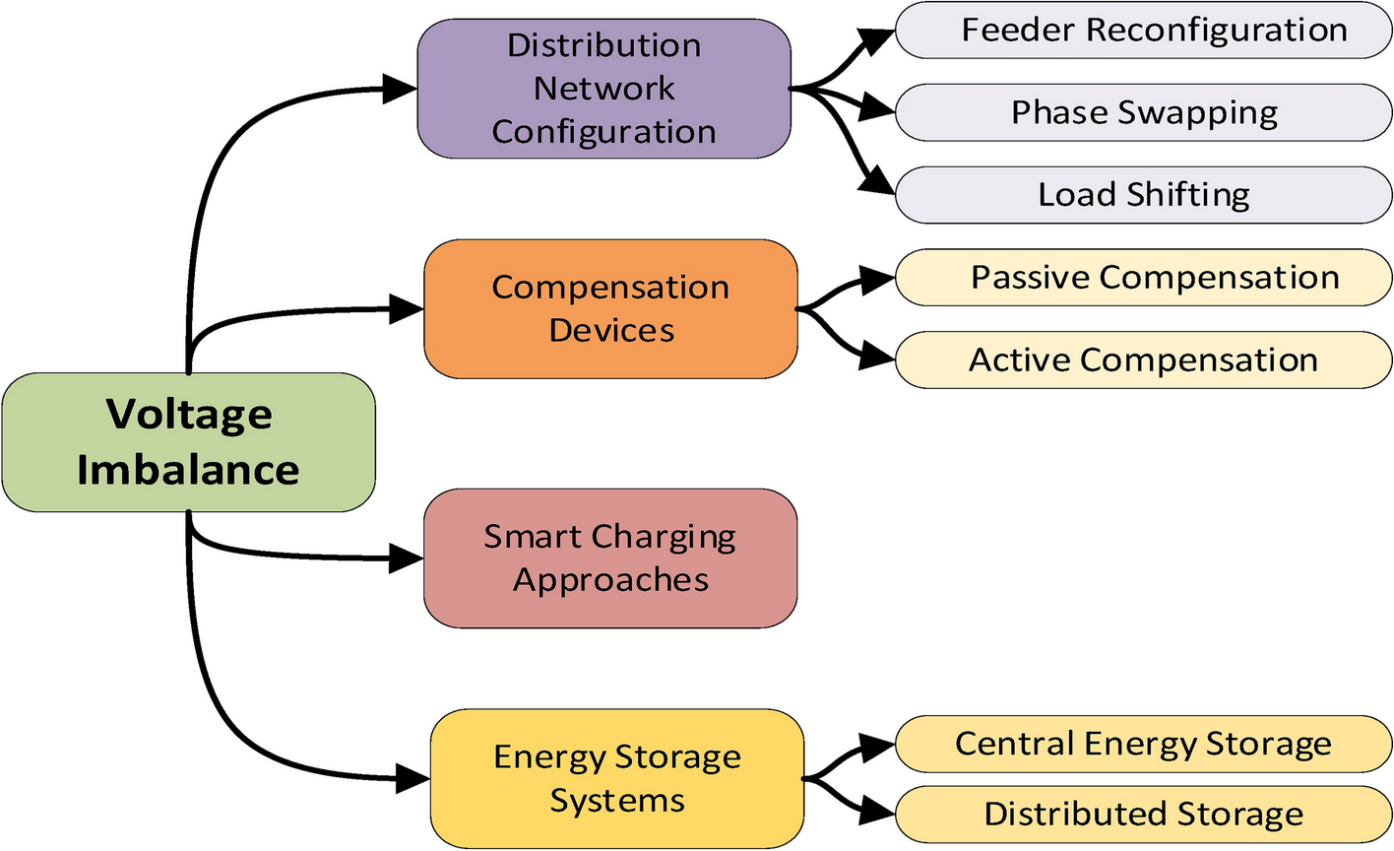
Voltage imbalance mitigation techniques.

High-capacity loads such as EVs, are typically connected to LV distribution causing three-phase voltage imbalances due to the charging and discharging action. These imbalances could lead to significant consequences such as: increased network congestion, increased peak load periods and harmonic distortions as well as noise generation due to unwanted pulsations leading to malfunction of protective relays. Besides, imbalances lead to more power losses, overheating to neutral lines, interference with power-line communication in addition to elevated overall power system operation and maintenance costs^59^. Several approaches have been proposed to tackle the adverse effects of voltage imbalances on the distribution grid. They can be typically categorized into four main groups. The first group implements Distribution Network Configuration which includes reconfiguration strategies based on solving multiobjective optimization problems such as feeder reconfiguration, phase swapping and load shifting techniques. Thus, they can reduce total power loss, imbalances and network reliability without procuring new equipment. The second group relies on mitigating the imbalance impact using compensation devices (equipment oversizing, passive and active compensation). Whereas the third group utilizes smart charging which can help in compensating both voltage fluctuations and imbalances^60^. Finally, the fourth group utilizes the usage of energy storage systems both in a centralized and decentralized manner to mitigate imbalance effects^61^. Figure 7 shows different voltage imbalance mitigation methods.

a. Distribution network reconfiguration strategies

Distribution network reconfiguration (DNR) strategies are often employed within distribution networks combined with other approaches, such as optimal placement of distributed generation (DG) units, to minimize voltage imbalance effects simultaneously and reduce system losses and operating costs. Reconfiguration techniques can be categorized into two types: distribution feeder reconfiguration (DFR) and phase balancing^62^. DFR generally focuses on modifying the topological structure of the network, such as changing candidate normally open tie switches and sectionalizing switches; thus, it is normally achieved at the system level. The phase balancing technique, on the other hand, focuses on the feeder level and can be implemented in two ways: phase swapping and load shifting. Table 7 summarizes the most recent work regarding DNR.

**Table 7:** DNR for voltage imbalance mitigation.

Ref	System	Purpose	Objective	Constraints	Control algorithm	RES & DG
^66^	Radial	Minimizing investment cost of distribution, including installing cost of SVCs and DGs	Minimize power loss, voltage deviation and imbalance	Power flow balance Equipment operating and voltage limits	Teacher Learning-Based and Comprehensive teaching–learning-based algorithms with compensation	DG
^67^	IEEE 33 Bus system	Dynamic reconfiguration minimizing power loss and voltage distribution	Power loss and voltage deviation minimization	Voltage limits Power flow constraints Transformer constraints	Non-dominated Sorting Genetic Algorithm with SVC	WT & PV
^68^	IEEE 33- and IEEE 69-bus	Optimal sizing and siting of DG and power factor with network reconfiguration	Active power and reactive power flow balance	Balanced generated power and load demand at buses Minimum reactive power injection DG and voltage limits	Water Cycle, particle swarm, Cukoo search, harmony search, fireworks algorithm	DG
^69^	IEEE 33 Bus system	Dynamic distribution feeder reconfiguration	Minimum Operation cost, energy loss, voltage deviation Minimum Energy Not Supplied	Line power limit Power balance Number of switching operations Maintain bus voltage limits Transformers limits	Particle Swarm Optimization Grey Wolf Optimization hybrid algorithm (IPSO-IGWO)	Wind
^70^	Microgrid	Optimal scheduling using batteries to increase system flexibility and RESs penetration	Minimize total operating cost	Power Balance Power generation, line capacity & BES limit	Lambda iteration, Lambda logic, Particle swarm, and Quadratic rotated conic programming	PV, wind, DG
^71^	Indian Utility 63 system	IoT-based hybrid optimization of DG’s location, size, and type	Maximize total customer benefit Minimize power loss, generation cost and emissions	Active and reactive power flow Power generation limits	Probability Based Incremental Learning with Firefly algorithm	DG
^72^	IEEE Standard-1547	Advanced DSM and energy management for optimal voltage and frequency	Minimize operating cost, grid emissions and PAR	Battery charging and discharging limits and operating voltage limits Maximizing line currents	GA optimization Improved PID H-infinity controller for voltage and frequency regulation	PV, wind
^73^	IEEE 33 system	Day-ahead network-reconfiguration model	Minimize operating cost, voltage deviations and power losses	Network, radiality, switching and security Constraints	Mixed-integer linear programming	Wind, PV, and diesel DG
^74^	IEEE 33 system	Energy management for hybrid systems including network reconfiguration and RESs scheduling	Minimize operation costs and energy losses	Voltage, angle, line, energy resource and converter capacity limits. Power balance and network reconfiguration constraints	Linear programming	PV, fuel cells, DG

b. Passive and active compensation strategies

As mentioned in^63^, passive approaches use capacitive compensation to address load imbalances. Conversely, the active approaches harness the capabilities of power electronic devices to inject active and reactive power, including line voltage regulators, reactive power compensator, distributed static synchronous compensator (DSTATCOM), dynamic voltage restorers (DVR), and unified power quality conditioner (UPQC) to rectify voltage imbalances. In^64^, reactive power injection is realized via two-layer optimization strategy designed for loss minimization while optimally placing capacitors in imbalanced test feeders. Despite being a cost-effective solution, it suffers from a few drawbacks especially in addressing voltage imbalances in cases where single-phase solar PVs generate high power, leading to active power imbalances. Dynamic Voltage Restorers (DVRs) are effective tools in addressing voltage disturbances by injecting series voltages, thus maintaining the magnitude and phase of load voltage^65^. Despite their benefits, DVR units are costly, and their performance may be affected by high-order harmonics present in the system^65^. The use of Unified Power Quality Conditioners (UPQCs) might be an alternative for mitigating both voltage and current issues^63^, however, their complexity and high cost may hinder their use. Alternatively, single-phase distributed energy resource inverters can compensate by injecting both active and reactive power without impacting the battery life or EV charging time. However, their efficiency with high imbalance levels is dependent on their power ratings and the number of installed units^63^. While DVRs, UPQCs, and various compensation strategies can effectively mitigate imbalances, limitations such as costs, complexity, and adaptability still hinder their usage.

c. Intelligent charging strategies

**Fig. 8:**
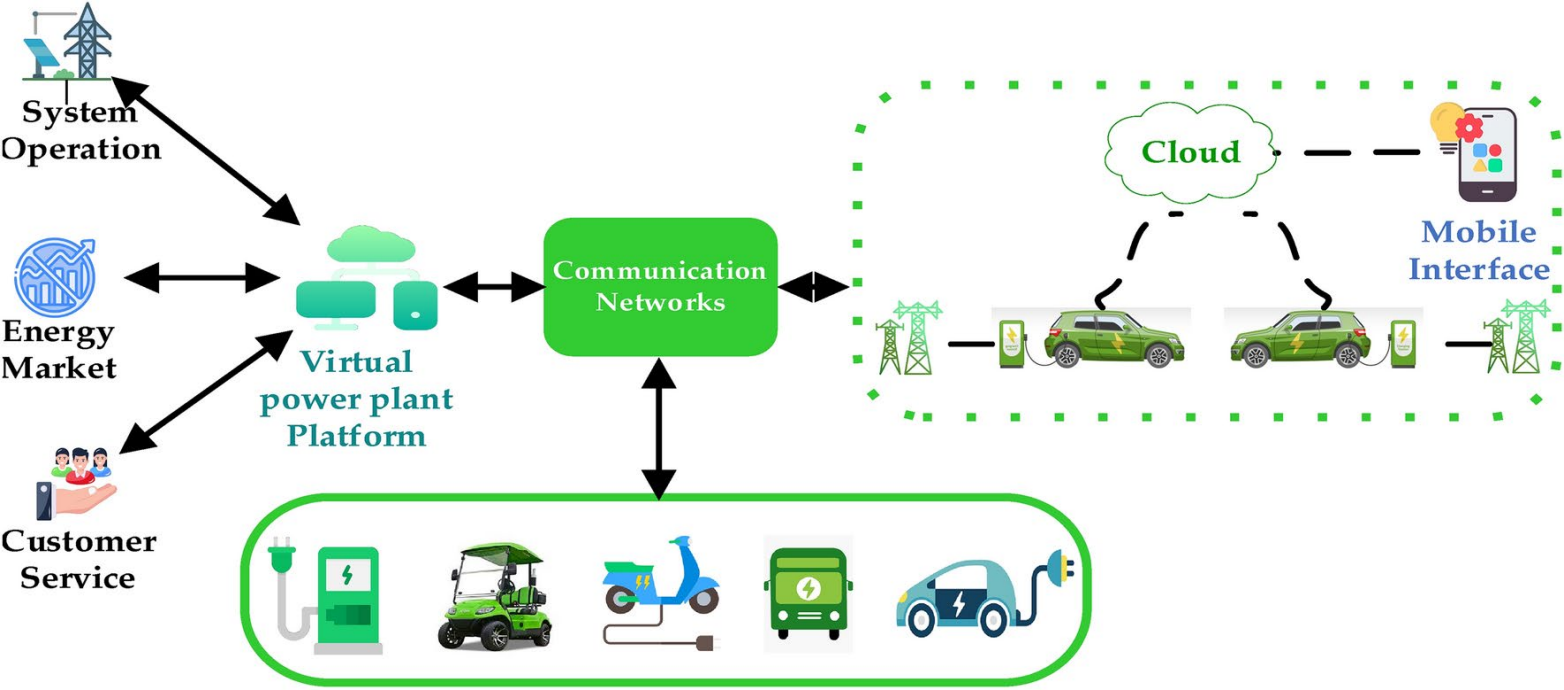
Intelligent charging strategies^75^.

Smart charging enables EVs to recharge during the most cost-effective time intervals, typically when the energy demand is low. Thus, the electricity demand is optimized, and the voltage deviations are reduced, aiding in flattening the voltage profile. Although smart charging yields advantages for both power utilities and EV owners, it necessitates additional infrastructure such as smart charging chargers, a communication framework and a processing system as demonstrated in Fig. 8^17^. This is achieved using centralized or decentralized strategies to improve distribution system performance by introducing the concepts of controlled EV charging and discharging, scheduling time of utilization (ToU), and controlling EV charging or discharging rate.

The smart charging of EVs significantly improves grid balance because of its ability to optimize energy distribution via real-time monitoring and management. This is achieved by integrating smart charging devices and allowing for dynamic responses to variable energy production and consumption and the number of online EVs^75^. Additionally, smart charging can mitigate voltage imbalance and neutral current overloading by coordinating EVs on the basis of the degree of grid imbalance^76,77^. The use of advanced control techniques and power electronic topologies in ultrafast charging stations additionally enhances the charging speed^78^; however, these methods require high infrastructural investments. Moreover, since smart charging systems completely depend on constant communication, coordination problems are likely to arise^69^. Furthermore, cyberattacks and privacy protection concerns are elevated, highlighting the need for more security protocols for data integrity protection^75^. Finally, voltage imbalance may be greatly affected by the increasing penetration of variable distributed energy resources onto the grid, which necessitates the use of additional inverter-interfaced DERs and energy storage devices^79^.

d. Energy storage system (ESS) strategies

The declining battery storage prices make ESS strategies an appealing solution for addressing both over-voltages and voltage imbalance. Centralized storage devices connected at the feeder ends can alleviate over-voltages when coupled with modified three-phase damping control which can charge/discharge the battery storage system by injecting or absorbing phase currents^78^. Conventional capacitor battery systems, on the other hand, have limitations when linear voltages are balanced since they may suffer from potential resonance and switching over voltages. Conversely, BESS can compensate for negative- and zero-sequence currents and reactive power, thus improving the power quality of the distribution grids^80^. In addition, BESS optimal control strategies can be applied in conjunction with PV systems for residential owners. Moreover, decentralized control strategies provide optimal performance for reactive power and voltage unbalance control with minimal impact on the battery owner’s needs. These strategies ensure that charging energy storage devices can take place without violating voltage limits and interfering with utility goals^81^.

#### Grid Instability

**Fig. 9:**
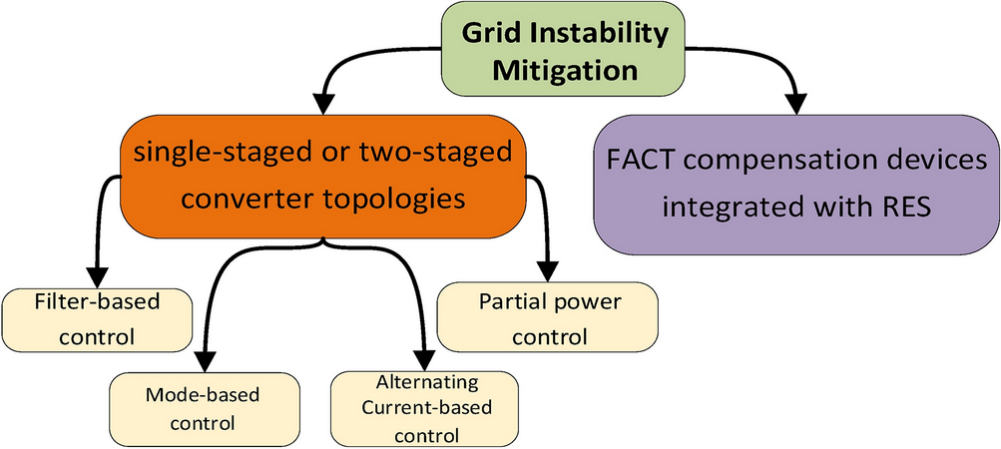
Grid stability mitigation techniques^85^.

Grid stability is defined as the ability of the system to respond to frequency, current, or voltage transient disturbances within an allowable margin of ± 5% from the rated values^82^. However, the connection of multiple high- fast charging stations to the grid makes it challenging to maintain regulated frequency and voltage at the point of common coupling. The growing presence of EVs on the grid network, especially with the use of fast charging stations, raises concerns about grid stability due to the significant amount of power drawn from the grid in the G2V mode, even with standard level-2 charging, often surpassing the typical peak demand of residential households^83^. Furthermore, EV owners tend to charge their vehicles after work, coinciding with the peak demand on the grid, resulting in a significant rise in peak system demand and posing more threats to grid stability. Grid instability can have notable impact on consumer services, existing infrastructural assets, as well as all network charging stations^84^. Improvements in grid stability can be approached via two schemes, as shown in Fig. 9, either via power converter topologies^85^ or through FACT compensation devices integrated with RES^86^.

a. Power electronics based on single- and two-staged control

Power converters (in both single and two-stage topologies) provide powerful tools for voltage regulation and voltage and frequency control abilities which are useful in maintaining low voltage-network stability. One of the primary benefits of employing power electronic converters to solve stability issues is the power flow fast and accurate control, which is necessary with standalone microgrids and during transitions between grid-connected and islanded modes^87^. Advanced control techniques, such as adaptive Virtual Synchronous Generator (VSG) control, improve transient stability by promptly responding to power imbalances^88^. This adaptability is advantageous in systems with high-RES penetration due to increased voltage oscillations^89^. In^87,90^ authors use a sliding mode-based control (MBC) with high adaptability to parameter variation in fast charger stations operation at reduced switching losses, yet with high computational requirements. The filter-based approach introduced in^91,92^ uses a control strategy based on hysteresis and voltage-oriented control for circulating current reduction and reactive power support. However, this method suffers from high control complexity. Moreover, the integration of power electronic topologies, such as DC-DC converters and multiport partial power processing converters (PPPC), in ultra-fast EV charging stations, allows higher power capacity operation and ensures compliance with grid connection standards^93^. However, the introduction of inertia support through VSG can induce power oscillations, which may lead to slower system’s response speed and reduced sensitivity to external disturbances^89,94^. While advanced control methods can adaptively change inertia and damping ratios to improve performance, they require precise tuning and can be challenging to implement effectively in actual real-life scenarios^94^. In summary, while power electronic control has several advantages in enhancing grid’s stability, it also presents challenges related to system complexity, tuning requirements, and potential adverse dynamics due to excessive virtual inertia. These factors must be carefully balanced to optimize the functionality and ensure reliability EV adoption and penetration.

b. FACT compensation

Flexible AC Transmission System (FACTs) are powerful devices that can offer a wide range of network support functions, nevertheless power system stability. Table 8 summarizes the most recent work for FACTs-based compensation topologies to solve EV to grid integration problems where D-STATCOM was applied in^95,96^ UPFC in^97,98^, SVC in^99^ and SSSC in^100^. In^101^, the efficiency of different FACTS devices (STATCOM, SVC, CSC and SSSC) were compared for voltage-stability where STATCOM was proven to be more effective than SVC and SSSC. FACTS were efficient in reducing the settling times and damping power oscillations when optimally tuned using algorithms like the Quasi-Oppositional Differential Search Algorithm (QODSA) in^99^ or Linear Active Disturbance Rejection Control (LADRC)^96^. The use of advanced optimization-based techniques has also demonstrated superiority in improving transient stability and reducing errors^97,98,100^. Moreover^95,99,101^, investigated how FACTs compensation with DG/RES integration can affect network stability and enhance power supply reliability. However, the integration of EVs and RES introduces complexities especially for real-time stability assessments, which requires complex control for effective operation^101^. Besides, the existence of different types of RES requires sometimes tailored solutions to disturbances, making the solution more complex^101^. Despite these technical advancements, limitations and operational challenges remain, particularly with respect to real-time stability control. Thus, coordinated actions among various controllable devices are needed to maintain optimal voltage profiles and system stability^101^.

**Table 8:** FACT- based compensation topologies for solving EV to grid integration problems.

Ref	Purpose	Configuration utilized	Control	Comparative study	Results	RES
^95^	Voltage profile improvement contributing to better stability of power system	D-STATCOM	GA optimization	–	Enhanced voltage profile Reduction of active power loss	DG
^96^	Disturbance rejection and stability improvement	D-STATCOM	Linear Active Disturbance Rejection Control (LADRC)	PI controller	Faster adjustment time Better reactive current tracking Stronger anti-interference ability compared to the PI controller	no
^97^	Damping out low-frequency oscillation through the coordination of FACTS with power system stabilizers	Unified Power Flow Controller (UPFC)	MGGP approach to optimize power system stabilizer parameters	Different operating condition and different control techniques	Minimum damping ratio Minimum time domain simulations Minimal time (less than a cycle) to estimate the key parameters compared to PSO and IWO	no
^98^	New damping controller for minimizing oscillations in single and multimachine power systems	Unified Power Flow Controller (UPFC)	Harris Hawks Optimization algorithm	Whale Optimization Algorithm (WOA) Grey Wolf Optimization (GWO) Cuckoo Search Algorithm (CSA)	Minimum peak overshoot Minimum settling time Minimum computational times	no
^99^	Improving transient stability of the power system	SVC	QODSA for designing an optimal damping controller	Conventional PSO optimized PID, DSA optimized PID QODSA based PID	Damped low frequency oscillations	PV
^100^	Design and optimize an SSSC controller to enhance power system stability	SSSC	MISO control approach using whale optimization algorithm for controller parameters optimizing	DE (Differential Evolution) PSO (Particle Swarm Optimization) based SISO controllers	Minimum errors Stability is maintained with rapid oscillations damping	no
^101^	Compares the efficiency of different FACTS devices for voltage-stability based on the calculation of PV curves	CSC SSSC SVC STATCOM	Modeling approaches	Compares the PV area criterion for different FACTS devices	STATCOM may be more effective than SVC and SSSC for voltage control	PV

#### Overloading

A low-voltage grid cannot be responsive if all EV owners constantly and without pattern charge their vehicles. Owing to simultaneous or uncoordinated charging, regional demand profiles may shift significantly. This can have a significant effect on local peak demand, thus overloading the network^2^. Overloading distribution network equipment such as transformers and cables results in their overstressing, shortening their lifespans, and requiring infrastructure upgrades. One of the most important and costly pieces of equipment in a power system is power transformers. Hence, correct transformer loading has been a key concern for power system operation. Different overloading mitigation strategies are introduced in literature which can be categorized into (a) Network and feeder reconfiguration plans, (b) RES utilization, and (c) Demand response and load shaping.

Investigating the EV penetration impact on distribution system overloading has been addressed in several case-studies in literature as illustrated in Table 9. It is concluded that there is a direct relationship between EV penetration and increases in loading conditions and peak demands. EV growth without proper charging coordination may cause overloading, increase peak capacity, grid losses and power grid stress during peak hours. Moreover, peak power capacity is a limiting factor over network infrastructure leading to stresses on infrastructure and accelerated degradation of network and equipment. This is concluded from case studies in California and Sweden, where increased peak capacity due to EV penetration resulted in stresses on infrastructure, leading to problematic issues^102,103^. This is also evidenced by a study in the USA, which additionally indicated that accelerated power transformers aged, particularly during peak hours^104^; however, smart and coordinated charging is important for peak demand reduction. This is evidenced by a study in Britain, where smart charging reduced peak demand^105^, and by studies in the USA, which indicate that charging management reduces power grid stress during peak hours^106^ and can significantly reduce transformer overloading^104^. Moreover, in Egypt, applying the FL-based valley filling approach prevents peak demand surplus^107,108^, whereas in Maldives, coordinated charging can reduce feeder loading^108^. In Brazil ToU and Real-Time Pricing (RTP) could reduce peak demand^109^ while in France, tariff-based dynamic smart charging systems proved their usefulness^110^. Meanwhile, one major challenge of EV growth in many countries is the need for further development of EV charging infrastructure which still seen as a potential barrier to further adoption. In Egypt and Maldives, the penetration level of electric vehicles is limited adoption due to infrastructural and economic challenges^107,108^. Moreover, in France, increasing the current ratio of EVs to charging points is needed^110^.

**Table 9:** EV penetration levels & overloading.

Ref	Country	Penetration	Peak load rise	Conclusions
^102^	Sweden	55%	Not specified	Peak power capacity is a limiting factor over network infrastructure which may cause problematic issues
^103^	California, USA	23.9%	10%	EV growth in California will lead to stresses on infrastructure leading to accelerated degradation of equipment
^104^	Western Kentucky, USA	10%	18%	Grid losses ranging between 40 to 62% during off-peak and peak charging periods. Distributed managed EV charging strategy was able to significantly reduce transformer overloading, thus reducing grid losses
^105^	Great Britain	100%	30%	The study shows that the GB transmission network can support 100% of domestic charging, with smart charging as a key solution for reducing the percentage of peak load to 9%
^106^	Tennessee, USA	1.2%	Not specified	Multi-charger framework with V2G mitigates power grid stress during peak hours
^107^	Egypt	50%	20%	EV can serve up to 96 residential consumers. A fuzzy logic-based valley filling approach may be able to accommodate the EV penetration without peak demand surplus. However, there are challenges facing charging infrastructure
^108^	Maldives	30%	4.4%	Coordinated charging may lessen the generation capacity requirements to 1.8% which will consequently reduce feeder loading. However, there are challenges facing charging infrastructure
^109^	Rio de Janeiro- Brazil	30%	11% to 18% in winter and summer	EV can reach up to 2140 consumers. ToU and Real-Time Pricing (RTP) may reduce peak demand between 0.3% and 1.6% in summer, and between 8.9% and 2.6% in wintertime
^110^	France	26%	30%	EV is projected to serve around 24.4 million individuals in France. The study highlighted the importance of tariff-based, dynamic smart charging systems as well as the V2G operation modes. Ratio of EVs to charging points is challenging

In conclusion, studies collectively suggest that implementing smart coordinated charging solutions may, in fact, increase the grid capacity and reduce power losses and grid stress. Moreover, the availability of public and private charging stations as well as the upgrading of infrastructure assets and equipment are recommended since they significantly promote higher EV adoption rates.

#### Harmonics

One of the challenges associated with EV battery charging is the potential high harmonic currents associated with converting the voltage of an AC power system to the DC voltage of an EV battery. Harmonic currents increase the losses and reduce the life expectancy of distribution components such as capacitors and transformers. The supply of harmonic current also creates harmonic voltages in the distribution network which can affect the power system’s loads and therefore must be maintained within specific standards. The majority of international standardization organizations have developed power quality standards which specify limits to harmonic pollution and its various effects, such as voltage swells and flickering. IEC 61000 and IEEE 519 are two of the most popular standards. As the number of EVs on the road increases, new standards to reduce harmonic pollution caused by EVs are introduced. IEC 61851, which is related to onboard chargers, is one of these forthcoming standards^111^.

The harmonic profiles of EVs include significant content of the 5th, 7th, 11th, and 13th harmonic components, which causes increased power losses and premature transformer failures; hence, they need to be mitigated^112,113^. In general, addressing harmonic impacts on distribution networks can be achieved via several harmonics’ mitigation strategies, as summarized in Table 10. This includes the utilization of hardware solutions such as power conditioners and filters (passive and active), software harmonic compensation approaches, or the integration of RES inverters^113^. Hardware solutions can achieve tremendous reductions in THD^114,115^. Furthermore, the use of PV inverters as active filters is an effective approach. This strategy improves the power quality by leveraging the switching frequency of PV inverters to compensate for higher-order harmonics^116^. Although hardware solutions and the integration of PV inverters can improve voltage quality and mitigate the impacts of harmonic currents, they require high initial investment and increase system size, cost and implementation complexity. Thus, the use of advanced optimization algorithms for effective harmonic compensation is a reasonable alternative^112,117,118^ but at the cost of increasing control complexity. Conclusively, while the implementation of different harmonics-mitigation methods can be resource intensive and complicated, their ability to address power quality issues makes them necessary components in smart grid systems.

**Table 10:** Harmonic mitigation strategies.

Ref	Solutions	Solution nature	Results			
^112^	A genetic Algorithm (GA) based approach was used to optimally reduce the THDs by coordinated dispatch of EVs and wind generators (WGs)	Software			Before	After
			THDv	Vab	5.91%	3.72%
				Vbc	6.80%	4.04%
				Vca	5.84%	3.73%
^114^	An improved proportional quasi resonant (IPQR) controller was added to block the path of the DC bus voltage ripple affecting the grid harmonic currents	Hardware			Before	After
			THDi	Grid Current 1	12.6%	3.3%
				Grid Current 2	11.8%	2.9%
				Grid Current 3	12.4%	3.9%
^115^	For harmonic suppression, Active Power Line Conditioners (APLCs) were tested	Hardware			Before	After
			THDi		12%	3%
^116^	PV system installed at the charging station where the PV inverter operates as an active filter to compensate for high order harmonics	PV inverter involvement			Before	After
			THDi		12.2%	4.1%
			THDv		11.4%	5.6%
^117^	Optimal harmonic power flow algorithm was proposed to determine the optimal dispatch of the harmonic currents from PV-based DGs to mitigate the impacts of EV harmonic currents	Software			Before	After
			THDi		15.27%	11.6%
			THDv		7.39%	4.5%
^118^	A Lyapunov-based proportional integral with anti-windup (PI with AW) control was proposed for harmonic compensation with minimum DC-Link voltage oscillations	Software			Before	After
			THD	Grid Current 1	7.15%	2.97%
				Grid Current 2	5.73%	3.15%
				Grid Current 3	3.15%	2.02%

### Beneficial technical aspects of V2G technology in electric grids

**Fig. 10:**
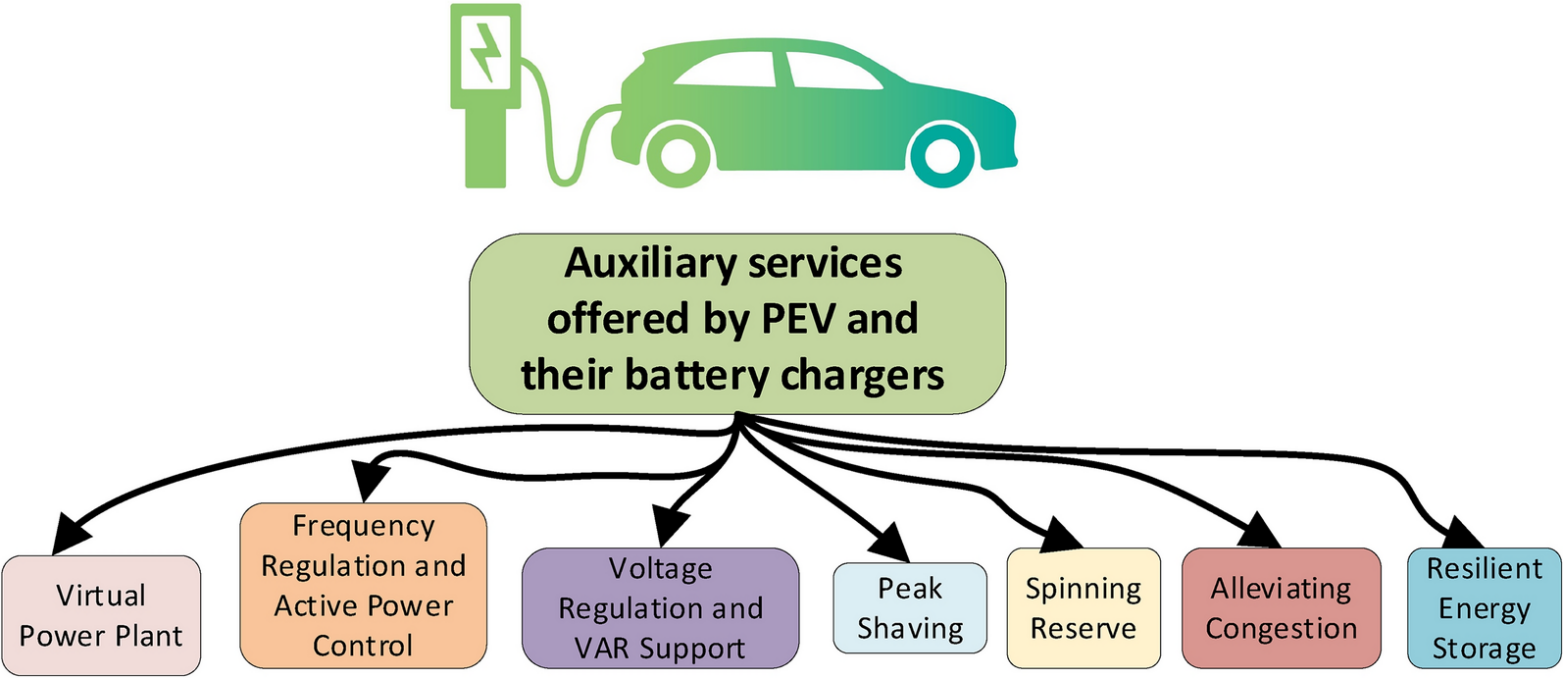
Potential V2G services.

Despite the negative impacts exhibited by the G2V mode of operation, numerous studies have demonstrated that efficient managing of EV charging can enhance power system efficiency, decrease operational expenses, and reduce the curtailment of RESs, particularly with the V2G feature. In V2G mode, charging can be switched to off-peak hours when electricity is less expensive or even negative. Revenues can also be raised by selling electricity to the grid at times of high demand for ancillary services or during peak hours. Additionally, V2G can help renewable energy integration by absorbing excess generation from variable sources and increase reliability by offering backup power in the event of grid disruptions or emergencies. Furthermore, controlled EV discharging offers several advantages such as offering reactive power assistance, regulating voltage and short-term frequency, reducing peak loads and load balancing as well as improving grid stability and harmonic filtering^117^. Plug in EVs (PEVs) and their battery chargers can be used for fast-responsive storage or as a distributed energy resource as EVs become increasingly integrated into the electrical grid^119^. In summary, bidirectional isolated chargers for EVs, via a proper control strategy, have the ability to offer auxiliary services as shown in Fig. 10^120,121^ and explained as follows:

#### Virtual power plant (VPP)

Virtual Power Plants (VPPs) are cloud-based distributed energy systems that interconnect energy generations and energy storage units within a complex power plant, enabling seamless energy management. The integration of EVs into VPPs plays a crucial role in stabilizing electricity demand. This is achieved through continuous monitoring, analysis, optimization, and trading of their energy output to counterbalance fluctuations in electricity generation from renewable energy resources. Small-scale energy facilities, on their own, lack the capacity to offer balancing services, but a VPP can effectively mimic the role of a large central power plant, providing similar services and ensuring redundancy^122,123^.

#### Frequency regulation and active power control

**Fig. 11:**
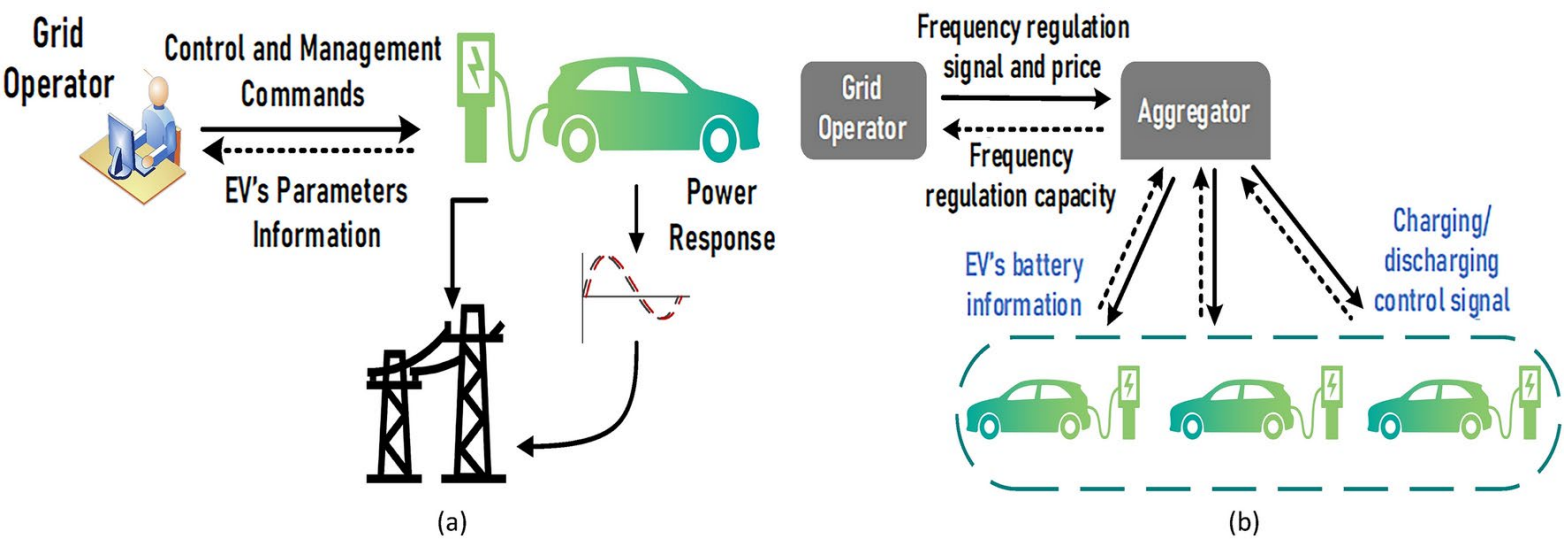
Frequency support systems (**a**) distributed and (**b**) centralized frequency structure.

Power system frequency regulation is an important indicator of active power supply and demand balance. Deviating from the specified frequency limits can lead to either load shedding during under frequency events or the disconnection of generation units in case of over frequency. Given EVs’ faster response, compared to conventional generation units, strategically managing EVs’ batteries charging and discharging presents a viable option for maintaining the power system frequency. This can be achieved via a centralized or distributed approach as shown in Fig. 11. Several dispatching strategies have been presented to assess the effectiveness of EV in their involvement in secondary frequency regulation, which can be typically divided into frequency-aware and economic-aware aspects as highlighted in^124^ and summarized in Table 11. The former primarily focuses on frequency stabilizing strategies (in both distributed and centralized modes), whereas the latter promotes the use of EVs for frequency regulation, increasing the benefits for both owners and aggregates.

**Table 11:** Frequency regulation strategies.

Ref	Modelling /optimization	Mode	Purpose	Horizon	Flow
^125^	Statistical	Centralized	Economic benefit	Day-ahead	G2V2G
^126^	Multi-objective optimization model	Hierarchical	Frequency regulation benefit	Day-ahead	G2V2G
^127^	Empirical model	Decentralized	Economic benefit	24 h	V2G
^128^	Multiple Integer linear programming	Hierarchical	Frequency regulation benefit	Realtime	G2V2G
^129^	Nonlinear Integer Programming	Decentralized	Economic benefit	Day ahead & Realtime	G2V2G
^130^	State space via Marcov transition matrix	centralized	Frequency regulation benefit	Realtime	G2V2G
^131^	Particle swarm optimization	Decentralized	Frequency regulation benefit	Day-ahead	V2G
^132^	Model Predictive Control	Centralized	Economic benefit	Real-time	G2V2G

#### Voltage regulation and reactive power support

Recently, a significant area of research has explored the ability of EVs to offer voltage and reactive power support when operating in V2G mode. In^133^, a unified strategy is proposed for voltage and frequency regulation within urban power grids by rapidly adjusting EVs charging or discharging power, in case of any deviation, particularly when the vehicles are parked. A comprehensive control approach for a bidirectional isolated EV charger, with the ability to offer auxiliary services, was suggested in^121^. This method focuses on off-board three-phase smart chargers for active and reactive power injection for grid support during voltage and frequency fluctuations. Using V2G technology through a network of charging stations, a hierarchical bi-directional aggregation algorithm was suggested in^134^ for the integration of EVs in the smart grid. By maximizing EV charging and discharging functionalities, the suggested algorithm predicts the power consumption and executes Day-Ahead load scheduling, thus stabilizing voltage and frequency. In^135^, an architecture for the cooperation between EV-sharing operators and Distribution System Operators was suggested for the provision of Ancillary Service in EVs. The designed system offers primary frequency control in a transmission grid and voltage amplitude regulation in a distribution grid. Finally, the authors in^136^ established a two-tiered dynamic gaming approach involving battery charging and swapping stations along with EVs to assist in grid node voltage regulation.

#### Peak shaving

The total grid system is under more stress as demand (load) increases; thus, “peak shaving” benefits the grid operator, end users, and environment in addition to providing an obvious economic advantage. This involves reducing power consumption to prevent spikes or deriving extra electric power from local power sources, such as a rooftop photovoltaic (PV) system, batteries or bidirectional electric vehicles. Peak shaving techniques produce an effective demand profile, which aids in improving system efficiency by enhancing power quality and lowering costs. However, the effectiveness of peak shaving depends critically on the quantity of EVs involved in the grid integration process. Efficient administration and optimization of the systems may also be impacted by the difficulty of coordinating charging and discharging of many EVs^137^. Authors in^138^ investigated into how the electricity and transport systems interact during concurrent peak times. Commuters who choose to discharge receive benefits but pay more for delays and change their departure time. Thus, a decision tree for the power system is designed to set the optimum discharge incentive and derive the necessary condition of the “win–win–win” scenario for EV drivers, power, and transport systems. Conclusively, lowering electricity costs in the form of rewards, incentives and financial compensation is essential in encouraging customers to take a positive step toward V2G technology.

#### Spinning reserves

The spinning reserve is the surplus capacity that may compensate for power shortages or frequency drops within a given specific time frame. Notably, “spinning reserve” is another auxiliary function that V2G technology may provide. However, having a sufficient number of EVs that are linked to the grid and have enough energy stored in their batteries to act as a spinning reserve is once again a difficulty^139^. However, in^140^, three different scenarios were considered: with V2G, without V2G, and with V2G plus a wind farm, where the latter greatly increased the grid’s reserve potential.

#### Alleviating grid congestion

When the amount of electricity consumed or the load on the grid increases, grid overload may occur, resulting in bottlenecks that prevent electricity from reaching consumer conditions, referred to as “grid congestion”^141,142^. This additional demand for energy would have to be met by the reserve power plant, which may result in higher costs and even higher demand at peak hours^142^. Moreover, transmission lines and system equipment may need to be upgraded to withstand this overload, thus causing a further increase in electricity costs. By using the energy from standby EVs, V2G can provide a solution for grid congestion, avoiding the need for pricey grid infrastructure changes. In^142^, a method in which power distribution factors (DFs) are employed to calculate how much energy a specific EV should contribute to reducing congestion was suggested. Nevertheless, with strong PEV penetration, uncoordinated charging of PEVs causes congestion. Thus, this issue was addressed in^143^, where a novel management method was proposed, and the SOC for PEV batteries was predicted via the gradient boosting regression tree method for optimal coordination of PEV charging to prevent congestion issues.

#### Resilient and sustainable energy storage

**Fig. 12:**
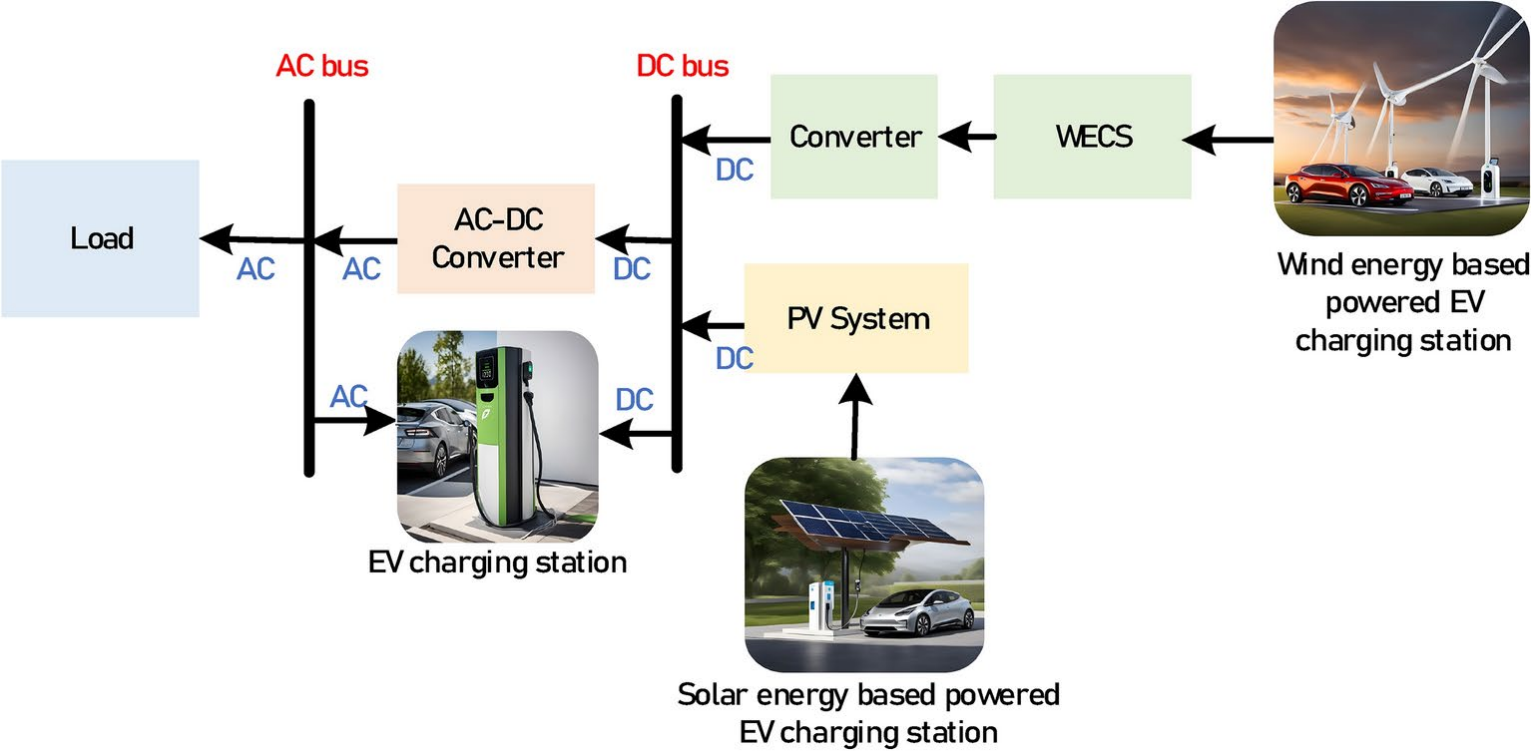
EVs storage capability for RESs power^147^.

The increased integration of RES into the power grid has led to intermittent energy production. When the amount of electricity produced by RESs outweighs the demand, the excess electricity can cause an imbalance in the system. Because of system congestion and mismatched supply and demand, RES curtailment, i.e. reduction of RES power production, is frequently utilized^144^. Although this act maintains grid stability, it raises operational costs of these RESs and reduces their efficiency. Hence, energy storage solutions play a crucial role in bridging this gap by storing excess energy when demand is low and delivering it when demand increases. If RESs excess power is used to fuel EVs, as shown in Fig. 12, this would be a very effective approach to store this extra power, thus enhancing grid resilience. V2G technology has the potential to provide a sustainable and resilient energy storage solution, thus V2G regulations that reduce RE curtailment are seen favorable^145^. Additionally, reducing curtailment can encourage investors to fund future renewable energy projects^146^.

### Technical challenges and research gaps

With the previously discussed benefits of EVs especially with smart V2G charging, a great effort is put towards closing knowledge gaps and tackling research challenges facing the adoption of this technology on a large-scale as discussed in this section.

#### EV battery handling

For EV battery manufacturing, the commonly used raw materials include but are not limited to lithium-ion, nickel, cobalt, copper, and graphite. In^148^, it was concluded that both Lead-acid and NiMH battery-based are uneconomical to implement in V2G techniques. However, to improve the EV battery lifetime and reduce its degradation, the following perspectives should be considered^12,13^.

- Since batteries are the most critical component in V2G technology, developing relatively affordable and safe battery energy storage systems (without overheating issues), such as lithium-ion batteries, is one of the market perspectives of EVs^19^.
- The EV battery life cycle is analysed by examining the battery capacity to provide the necessary torque to run the EV wheels and to provide adequate power back to the distribution grid in^149^. The 60 kWh Li-ion batteries launched for second-generation EVs should feature a state of health above ~ 75% to sufficiently run the EV. These batteries can back almost 350–500 km drive range for approximately 14–20 years. Hence, second-generation EV batteries are adequate for EV and V2G implementation and seldom require replacement. However, the studied lithium-ion battery lifetime model can be extended to estimate the battery degradation level as the temperature increases, the uncontrolled state of charge, the unscheduled battery discharge/charge cycle, and the depth of discharge, as well as to estimate the associated increase in EV charging and energy/power fade. Conclusively, one of the main challenges for EV penetration is its limited driving range; thus, EV battery deployment must be precisely planned to realize both transportation and power system objectives and needs.
- The long-term operation of V2G EVs with high battery capacity increases the depth of battery discharge and stresses the powertrain. During bidirectional power flow between the vehicle and grid, EV charging and discharge should be optimally controlled, or the battery lifetime and capacity level should degrade. Compared with other typical charging methods, optimized charging control makes the battery last longer for a regular EV run.

Thus, in the V2G technique, multiple object optimization algorithms should be considered to properly schedule the EV fleet to reduce battery degradation and make the system more economical.

- Employing climate control methods and improving the included ventilator system is an appealing research point since this helps to improve the battery lifetime of EVs; thus, expanding the driving range can be improved^150^
- High-frequency current peaks, which are supplied during motor runs, should be mitigated since they can result in fast battery degradation^151^. To address this issue, the application of supercapacitors along with EV batteries can be promising^152^.
- Improvements in battery technology should be expanded to deliver a better range and increase the safety, flexibility, and efficiency of EV technology. This can be achieved by developing unique anode materials or electrolytes for Li batteries, in addition to creating solid-state batteries and batteries that replace Li ions with Na or K ions to improve their safety, energy storage, and efficiency^14^. All these developments in the production of organic Li batteries, along with safe repurposing and recycling of Li batteries, can make EV technology greener, cleaner, more robust, affordable, and safe.

#### Charging/discharging of EVs batteries

The Smart and coordinated charging and discharging of EV batteries, featuring V2G technology, is crucial for the efficient operation of EVs and for reducing the battery degradation time as well as minimizing the previously discussed negative impacts resulting from EV excessive integration to grid. This is done using power-electronic battery chargers associated with charging control techniques which in turn raises several interesting topics that should be considered^15,18^.

- EV Battery chargers

The EV battery charger is a power electronic converter that offers a bidirectional interface between the EV and the grid to charge the EV battery with the required capacity or feeds the power back to the utility, following all power regulations and standards. Fast power transfer with minimal losses is essential in V2G technology. Therefore, research related to EV chargers should focus on developing topologies that handle the complexity of modern power with the following capabilities^15,153^.

- Robust and high-efficiency topologies with a bidirectional power flow capability and fast charging feature
- Bidirectional converter topologies must be effective in terms of the power factor, power consumption, number of filters, number of switches, and THD.
- The enhanced efficiency of advanced converter materials is a promising topic where wide bandgap (WBG) devices made of SiC (silicon carbide) and GaN (gallium nitride) are attracting attention. This is related to the fact that they operate at high frequencies and temperatures with lower semiconductor losses, thus achieving a reduced converter size and high power density^15,153^.

b. Coordinated charging/discharging control

The effective coordinated control of power electronic converters assists in supporting the bidirectional power flow between the vehicles and the utility grid, handling grid-integration requirements and standards as well as minimizing the detrimental impacts from the integration of EVs. Thus, it is necessary to develop optimal EV charging/discharging techniques that incorporate multi-stack optimization to maintain a charge–discharge equilibrium, reduce grid stress, improve frequency regulation, and reduce costs as well as enhancing battery lifetime^19,36^.

c. Planning of public charging infrastructure

Currently, the greatest barrier preventing V2G from expanding is the lack of EV charging stations. Hence, modifying existing networks and upgrading them to accommodate electric vehicles is mandatory, as is adopting new large-scale charging stations in the near future, streets, highways, workplaces, shopping centers, etc. Hence, many studies should be conducted in this area to introduce upgrading options and plan future infrastructure, considering the optimal location and capacity planning of EV chargers and grid integration concurrently^154^. Moreover, for successful implementation of a completely modernized V2G charging infrastructure, it should be equipped with adequate smart devices to be user friendly and reduce customers’ range anxiety. In addition, fast charging and high-power facilities can be an advancement in V2G systems^153^. Notably, EV charging stations couple both the utility system and the transportation system; therefore, both must be taken into account during EV charging infrastructure planning. Nevertheless, research in this area is challenging since EV infrastructure planning studies require real data from both the transportation sector and power system, which varies across countries^18^.

#### Coordination between transmission and distribution systems’ operators for providing EV services

As previously discussed, EVs can provide many local and system-wide services to different power system parties (i.e., transmission system operator (TSO), transmission system operators (DSO), and loads such as buildings or homes). However, system-wide services offered by EVs may affect the distribution system at which they are connected causing conflict of interests between TSO and DSO. For example, the frequency regulation TSO service offered by EVs requires continuous charging and discharging operations which can result in overloading of distribution network components, phase unbalance, etc. Hence, coordination between TSO and DSO is important to guarantee reliable and cost-effective services with minimal impact on the distribution system. However, this issue has rarely been studied in literature and should be considered in future research^18^.

#### Vehicle-to-everything (V2X) communication system

A vehicle-to-everything (V2X) communication system is a connected mobility concept in which the EV and any other systems inside and outside the vehicle are linked to the internet. This allows a vehicle to communicate with different entities though specific technologies such as V2I (vehicle-to-infrastructure), V2N (vehicle-to-network), V2V (vehicle-to-vehicle), V2P (vehicle-to-pedestrian), and V2D (vehicle-to-device). This mobility technology should be widely adopted because of its numerous benefits^16^.

- This increases operation and efficiency in power grids.
- By reducing traffic congestion (by introducing a V2V facility in 20% of vehicles), traffic congestion can be reduced by 30%).
- This increases EV users’ satisfaction and revenue (by accessing traffic jam conditions and charging prices at different stations through V2X, users can choose the best possible option for them at any time).
- Improving the driving experience and increasing passenger safety and comfort.
- Overcoming the communication barrier of autonomous vehicle technology has led to rapid development in which a decision can be made by the vehicle itself without the help of a user.

#### Communication protocols and cyber protection

The successful implementation of V2G requires a reliable, real-time communication system between control centers, aggregators and EVs to secure the transfer of information across the power grid, EV supply equipment, charging infrastructure, and end-users. The aggregator sends data about the EVs at station, station location, charging details, power level, etc. to the control center which takes the accurate control decisions and sends the charging rate and time as well as power commands back to the aggregator which in turn sends charging details and power commands to EVs^15^. Considering this large amount of information to be transmitted as well as EVs mobility and fast response requirements, V2G communication demands efficient communication protocols and fast authentication and encryption/decryption which is still a challenging aspect that needs to be questioned.

**Fig. 13:**
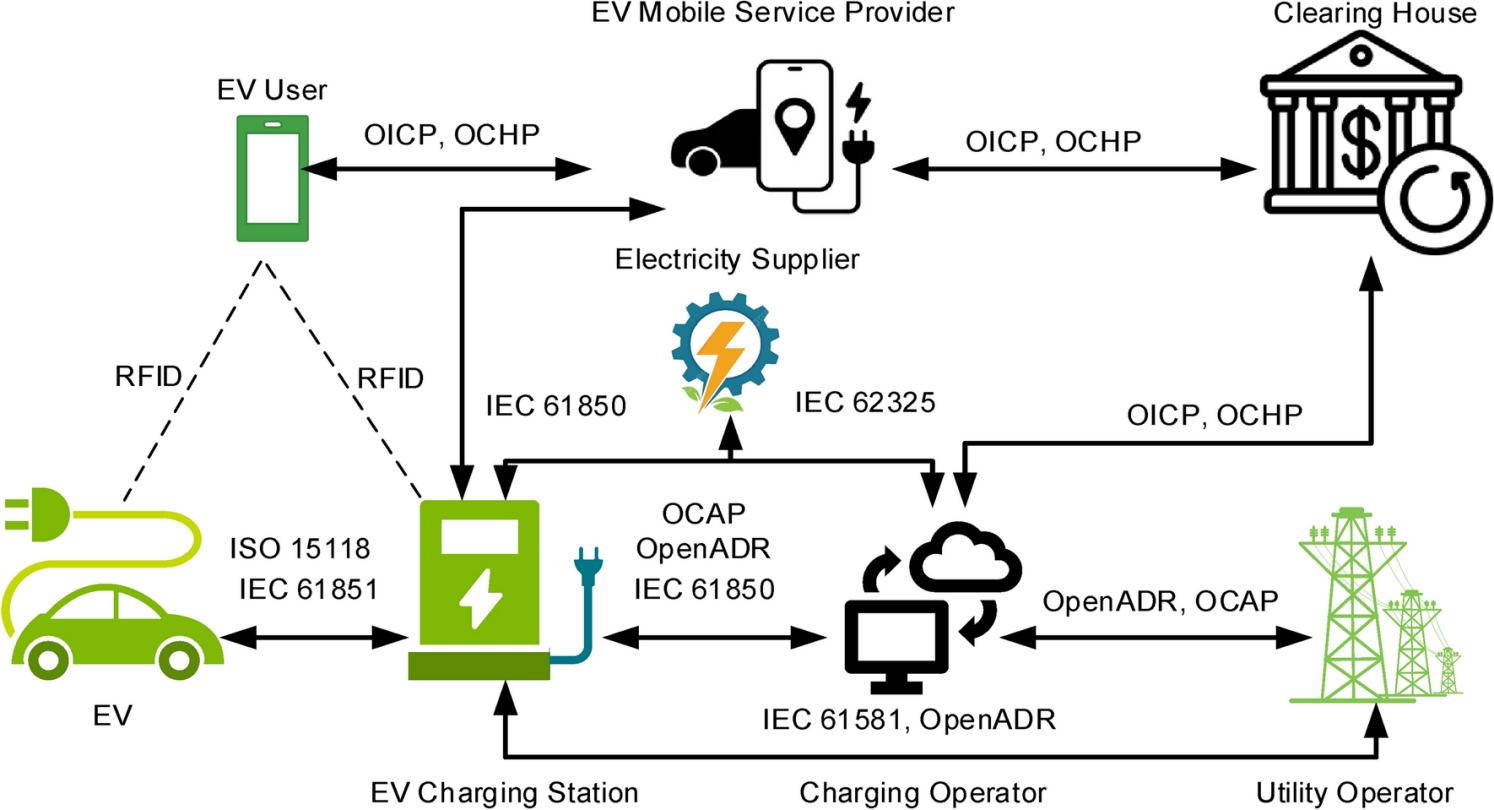
Protocol architecture for the EV-charging infrastructure.

Figure 13 shows the most common communication protocol architecture of the EV-charging infrastructure which involves various protocols designed to assure maximum compatibility and performance across all EV ecosystem. It is clear that the electric mobility system is categorized into different layers to efficiently manage all cyber-physical aspects of EV charging. Accordingly, charging levels can be divided into two levels of integration, (a) between charging point and energy suppliers, and (b) between EV and charging point. Open Charge Point Protocol (OCPP) and Open Automated Demand Response (OpenADR) are the most used standards in practice used for integrating charging points with energy providers. OCPP allows communication between charging stations and centralized management systems, thus enabling remote monitoring and charging control. OpenADR, on the other hand, allows for demand-response actions that can help in grid load balancing and ensures cross-domain information exchange with grid management system^111,155^. In addition to OCPP and OpenADR, IEC 61,850 allows seamless communication and compatibility in power utility automation and DERs control. For communication between charging stations and EVs, standards such as ISO/IEC 15,118 and IEC 61,851 support smart charging and V2G functionalities and facilitate the use of EVs as temporary energy storage units for grid support^111,155^.

Roaming protocols are important in assuring connectivity between different charging networks and service providers^111,155^. Roaming allows EV users to use different service providers to access charging stations without the need for multiple subscriptions. Open Clearing House Protocol (OCHP), and Open InterCharge Protocol (OICP) are examples of commercially used roaming protocols that provide a standardized framework for data exchange, pricing information, and authentication. Despite the increasing efforts of policymakers and regulators to adopt the use of open standards, the market remains fragmented with various incompatible roaming protocols thus hindering the EV integration and widespread adoption of BEVs^106^.

Moreover, another important aspect in this area should be considered which is the required protection in V2G systems against cyber penetration and threats associated with blockchain, AI, and IoT connectivity^12^. To prevent cyber security concerns, a mutual, reciprocal authentication technique can be developed in which the EVs, connected to the aggregator, are to be registered and checked for authenticity before initiating the charging/discharging operation. Moreover, EVs and aggregators can be designed with unique identification secret keys to filter out any malicious data flow across the network. This will ensure the confidentiality of user information, charging/discharging routines, etc.

### Technical key-findings, challenges and recommendations for EV penetration

The previously discussed technical impacts of EV integration into grids are summarized, as are challenges limiting their adoption growth, as shown in Table 12. Then, relationships between key findings and research gaps are identified, and recommendations are drawn to enhance EV performance, mitigate their limitations and promote their penetration in the grid.

**Table 12:** Technical key findings, challenges and recommendations.

Impact	Key findings	Challenges and research gaps	Recommendations
Voltage variation	VAR management is effective for minimizing voltage deviations	Centralized control may lack reliability and flexibility needed for localized issues	Integrating decentralized control with centralized one offer improved localized decision-making, leading to better reliability while maintain enhanced voltage regulation
	Centralized control is effective in achieving voltage regulation		
Grid imbalance	Optimal network reconfiguration using advanced optimization techniques reduce power losses and voltage imbalance DG units’ integration cause further reduction in losses and imbalance	Despite feeder reconfiguration techniques can help alleviate system-level imbalance, they are generally less effective in addressing significant phase imbalance at the feeder level	Implementing phase balancing either via phase swapping or by redistributing loads among the different phases to solve phase imbalance problems
	Passive and active compensation can solve imbalance conditions	Despite, its cost-effectiveness, passive compensators limitedly solve grid imbalance	Active compensators as DVRs & UPQCs, solve imbalance yet at more cost and complexity, thus cost-effectiveness enhancements are required
	BESS compensates for negative and zero-sequence currents	Limitations regarding BESS: cost, lifetime and deployment	Advancements in BESS to reduce their cost and degradation are required
Grid stability and reliability	Smart charging technologies and grid control as well as communication protocols enhance the automation and self-healing capabilities of the network, thus enhancing grid stability	These schemes need significant infrastructural investments Reliance on communication makes it vulnerable to failures in coordination and possible risks of cyber-attacks	Upgrade charging infrastructure to house these technologies Apply security protocols to protect data integrity and privacy
	Power electronic (PE) topologies and FACTS can significantly improve grid transient stability and reduce errors when optimally tuned as well as improve grid balancing conditions	Despite PE advancements, still operational limitation, tuning requirements and complexity challenges exist, particularly with real-time stability control when integrating RES with EVs	Complex coordinated control and precise tuning actions among various controllable converters and FACTS are needed for effective operation and to maintain optimal voltage profiles and system stability
Harmonics	Hardware solutions as Filters and Power line conditioners can significantly improve voltage quality and mitigate current harmonics Additionally, integration of PV inverters and optimization techniques can realize reasonable THD reductions	Hardware solutions and PV inverters require high initial investment and increase system size, cost and implementation complexity Oppositely, software solutions increase control complexity	Although implementing harmonic mitigation techniques can add to system complexity, their ability to address power quality issues makes them a necessary component of the smart grid
EV high penetration and overloading conditions	EV high penetration in grid increases peak load capacity and result in overloading conditions EVs penetration is limited by its driving-range where EV battery system is the most crucial device	Peak power capacity and overloading impose stresses on infrastructure causing devices accelerated degradation Need for further development of EV charging infrastructure EV battery systems face cost and degradation challenges	Smart and Coordinated EV charging strategies is crucial for reducing peak loads, minimizing power losses, and maintaining voltage stability Upgrading infrastructure and equipment is important with high EV penetration Optimized charging scheduling make the battery last longer for a regular EV run, reduce its degradation and make system more economical
V2G technology	Benefits of V2G Technology Virtual power plants (VPP) Frequency and voltage regulation, Spinning reserves Peak Shaving Increasing grid resilience and reliability Alleviating congestion Renewable energy use	Barriers of V2G Technology EV batteries production, handling and disposal EV batteries charge/discharge scheme EV batteries charger topology EV Charging infrastructure System-services offered by EVs may affect the distribution system at which they are connected	EV batteries deployment must be precisely planned and improvements in battery technology should be expanded to become more safe, clean, reliable and efficient Optimized charging scheduling and coordination Develop robust and high-efficiency battery charger topologies Upgrading and expanding charging infrastructure and equipment Coordination between TSO and DSO is needed to guarantee reliable and cost-effective services
Communication system	Successful implementation of V2G requires a reliable, real-time communication system between control centers, aggregators and EVs to secure information transfer Vehicle-to-everything (V2X) communication system should be widely adopted for its benefits Roaming protocols assure connectivity between charging networks and service providers	V2G communication demands fast authentication and encryption/decryption V2G communication systems may face limitations against cyber penetration and threats with blockchain, AI, and IoT Market remains challenged with various incompatible roaming protocols, hindering widespread adoption of EVs	To prevent cyber security concerns, a mutual, reciprocal authentication technique can be developed EVs and aggregators can be designed with unique identification secret keys to filter out any malicious data flow across the network and ensure confidentiality of user information and charging /discharging routine, etc Increasing effort of policymakers and regulators to adopt the use of open protocols and standards

## Other impacts (regulatory/economic/social/environmental), related challenges and recommendations

To study other aspects rising from EV penetration into the grid, this section presents other impacts that are crosslinked together including regulatory (political), economic, social and environmental ones in addition to their respective challenges, research gaps and potential recommendations as summarized in Tables 13, 14, 15 and 16 respectively.

**Table 13:** Key findings, challenges and recommendations for regulatory and political impacts.

Impact	Key findings	Challenges	Recommendations
Government policies and incentives	Government policies and incentives have been found to significantly improve EV adoption and V2G regulation	Despite existing policies and incentives, still more standardizations and regulations are to be established and more motivations to be offered to enhance EV penetration and V2G use	Governments and policy makers should: Establish a uniform national technical standard in the field of V2G Offer tax reductions and electricity cost discounts to EVs’ users
Infrastructure availability and quality	Availability of parking facilities, especially public charging stations, positively impacted EV wide-spread and V2G adoption	Upgrading the electricity grid to handle increased EV demand and house advanced V2G technology Upgrading and expanding charging infrastructure is still challenging	Local governments should: Put a roadmap to employ V2G and set infrastructure planning processes with cross-disciplinary groups Standardize charging scheduling per operation level V2G modeling, capacity forecasting, performance analysis and V2G impacts investigate on grid
Collaboration and partnership with Corporates and stakeholders	Collaboration and partnerships of governments with other organizations, corporates and stakeholders are critical for promoting EV	Establishing agreements with private sectors and investors in the field of EV adoption is still not taken into account by many countries	High upfront costs, lack of charging infrastructure, and consumer education and awareness can be fulfilled by establishing agreements with investors Sharing resources and expertise and offering alternative financing options by organizations to facilitate the transition to e-transportation
Urban or rural location	Urban users are more likely to have EVs due to the ease of accessing charging stations	Rural users face barriers towards EV adoption due to the limited access to charging infrastructure	Governments and policymakers should expand charging infrastructure in rural areas and incentivize rural consumers to adopt EVs

**Table 14:** Key findings, challenges and recommendations of economic Impacts.

Impact	Key findings	Research challenges	Recommendations
Initial cost	Compared to ordinary vehicles, EVs with V2G are more expensive due to their high initial costs, yet reduction in operations and emissions can be achieved in regions with high fossil fuel taxes and low electricity prices	Despite EVs long-term savings, their total cost of owning (TCO) play an important role in consumer decision-making since the initial cost of EVs presents a significant barrier to their adoption	Direct research efforts towards simplifying V2G technology, reduce its initial costs and decrease EV batteries cost and degradation
Economic acceptance	Economic factors (as governmental subsidies, manufacturer’s cash incentives, total cost of owning (TCO) a BEV, fuel costs and battery prices) affect EVs economic acceptance and tend to close the price gap between traditional automobiles and EVs	Still many governments lack robust subsidies and incentives’ policies thus affecting EV acceptance and adoption Limited literature considering factors like fuel consumption variations for urban and highway trips and finance interest rates which should be addressed and analyzed	Subsidies benefit the vehicle-manufacturers as they lead to increased production and sales of BEVs, thus boosting economic activity Learn from countries which witnessed a noticeable shift to e-mobility as Norway owing to robust incentive programs, including significant tax breaks, reduced parking fees and access to bus lanes making EVs more affordable
V2G commercialization	EV-owners’-based studies reveal that there are other potential barriers against V2G adoption, the most important of which is consumer unawareness of grid integration benefits as well as their concerns of privacy and loss of control. Thus, to accelerate its adoption, V2G commercialization is mandatory	Challenges in V2G market exist as lack of a comprehensive compilation regarding types of EVs in the market, absence of a solid V2G business model and the shortage of governmental incentives and stakeholders as well as no serious steps are taken towards V2G commercialization	Implementing new e-trading schemes, enabling incorporation of RESs and setting special tariff structures for co-location of EVs and RESs to increase the revenue of EVs’ owners from V2G Creating proper awareness among EV owners Development of economic viability metrics and models for charging stations infrastructure Benefits of V2G technology is based on collaborations, funding, technology maturation

**Table 15:** Key findings, challenges and recommendations of social impacts.

Impact	Key findings	challenges	Recommendations
Social perception	V2G technology serves the community in many social aspects including public health, operating cost savings and job creations	Despite these benefits, the high upfront costs of EVs make it only affordable to high-income users leading to social inequalities Other barriers affecting public acceptance and adoption rates of EVs include those related to changes in transportation habits and concerns about charging availability and anxiety range	Developing affordable EV models by manufacturers and providing financial incentives and tax credits by government to low- and moderate-income consumers Developing a robust network of charging stations is essential to alleviate range anxiety and prompt social acceptance and
Awareness	Awareness of EVs benefits are critical factors for EV market growth	A lack of awareness and understanding of EVs benefits is a significant barrier to its adoption	Holding trainings, educational programs and public awareness campaigns by governments to increase user familiarity and acceptance to EV technology
Marketing and advertising	Marketing and advertising can motivate stakeholders for financial investments in the EV context	Limited marketing efforts in the area of V2G impose barriers against large investments in this sector	Marketing organizations and advertising campaigns should open new business contexts for EV products and services

**Table 16:** Key findings, challenges and recommendations of environmental impacts.

Impact	Key findings	Challenges	Recommendations
Promotion for clean mobility	Shifting from fossil fuel vehicles to e-mobility reduce emissions besides enhancing energy security by lessening reliance on fossil fuel which is an appealing factor for consumers Another influencing factors on consumer intention to use EV is cutting the costs associated with health problems related to air pollution	Still in many communities, no broad knowledge on EV environmental benefits and no penalties are set by government on high-emission vehicles, thus no reasonable efforts are put to promote EV adoption among consumers	Governments are advised to; Focus on promoting the use of EVs to reduce environmental pollution through awareness campaigns and education to encourage user responsibility and green behavior Implementing taxes on high-emission vehicles and offering incentives for EVs Raise the fuel oil tax or launch the carbon tax to promote the relative competitiveness of EV
EVs’ battery production and disposal	With high penetration of EVs, the demand for batteries is increasing, thus environmental concerns can arise and should be studied	Batteries production process involves a lot of chemical procedures that are dangerous to the environment and can lead to health hazards Materials used in EV batteries are challenging to recycle and careless disposal of these batteries can be toxic and may lead to fire hazards	Direct research towards organic Li-batteries production, along with safe repurposing and recycling of Li-batteries can make EV-technology cleaner and more affordable, and safe Have well-defined, environment- friendly end-of-life strategy for the batteries Consider second-life batteries reusing
Divided opinions	Concerns about EV batteries production, disposal and recycling may lead to divided opinions on EVs’ environmental merits	Despite the potential for EVs in CO_2_ emissions reduction, their environmental benefits are often doubted by consumers, who doubt EV batteries capacity and are concerned by high battery cost and their production and disposal risks	Governments and industry manufacturers should allocate research resources for battery technology development to help alleviate customers worries of EV adoption

### Regulatory/governmental aspect

Many studies have investigated the influence of this impact and its future directions towards EV adoption involving the following points:

#### Government policies and incentives

Government policies and initiatives, including different types of regulations and incentives such as standardization, tax credits, and subsidies, have been found to significantly increase EV adoption^22^. For a seamless transition to e-mobility, the IEA policy document on global V2G offers outlines of existing policies, along with rules and recommendations^156^.

Existing policies among many countries include the following^15^:

- Incentives and regulations implemented to promote the adoption of EVs and smart charging.
- Incentives for charging infrastructure development linked to renewables.
- Special tariff structures for the colocation of EVs and renewables.

However, to improve V2G implementation and increase its penetration with minimal challenges, the following recommended policies and actions should be discussed and considered^36,154^.

- A uniform national technical standard in the field of V2G should be established to facilitate development and implementation and standardize the interconnection process as much as possible.
- Offering tax reductions and electricity cost discounts to EVs on the basis of aggregator policies for participating in V2G is one of the most motivational strategies and provides a possible solution to future challenges^153^
- A climate change tax credit, which is based on alternative fuel refueling property, should be expanded to cover V2G.

Overall, these findings suggest that government policies and incentives can play crucial roles in supporting the EV market.

#### Infrastructure availability and quality

Several studies analyzed the impact of infrastructure availability and quality on EV market growth and V2G adoption where it was noted that areas with more accessible charging stations had higher EV adoption rates. However, upgrading the electricity grid to handle increased EV demand and house advanced V2G technology was crucial for promoting EV penetration^22^ Thus, the following is recommended by IEA^156^.

- States and local governments should be encouraged to deploy V2G and a road map to be established for making infrastructure planning processes at a national level with the help of cross disciplinary groups.
- Charging scheduling algorithms must be designed and standardized according to operation levels.
- Standardization of integration to RES to tackle their intermittent scalability and economic feasibility
- Work is required in V2G modeling, capacity forecasting and transient and steady-state performance analysis as well as analyzing V2G grid impacts on the grid and disseminating the results to the wider community.

#### Collaboration and partnership with corporations and stakeholders

Establishing agreements and collaborations between the government and private organizations can greatly support EV market growth by achieving the following^22,157,158^.

- Various challenges, including high upfront costs, a lack of charging infrastructure, and consumer awareness, are addressed.
- Share resources and expertise to accelerate the transition to electric mobility.
- Offer business model innovation and alternative financing options for promoting EV adoption.
- Corporate social responsibility can motivate organizations to promote EV adoption as part of their sustainability goals by offering incentives to employees and providing education and training to increase employee familiarity with EVs.

#### Rural versus nonrural locations

Location can also play a role in EV adoption where users in urban areas may have better access to charging stations and may be more likely to adopt EVs than rural users^159^ The latter may face limited access to charging infrastructure which sets a challenging barrier for EV penetration in rural areas. Therefore, governments and policymakers can address this by expanding charging infrastructure in rural areas and incentivizing rural users to adopt EVs.

### Economic aspects

Hereby, the economic impacts affecting EV market growth, as discussed in literature, are highlighted in addition to some recommendations to prompt their market growth and acceptance.

#### EV market

According to the IEA, there are approximately 10 million EVs on the road globally. The worldwide V2G market size was valued at US$ 1.77 billion in 2021 and is predicted to reach approximately US$ 17.43 billion by the year 2027. As of 2023, China leads with the most publicly accessible EV chargers of approximately 2.7 million chargers, followed by the USA and Netherlands. Norway, Iceland and Sweden, on the other hand, have the highest EV market share. These nations are facing EV adoption due to their strong governmental incentives, extensive charging infrastructure, and public policies targeted at reducing carbon emissions and promoting sustainable transportation and e-mobility^1^.

#### Economic acceptance

Compared with conventional vehicles, EVs with grid integration featuring a modified charging structure can be more expensive due to high initial and investment costs (more complex implementation and costly bidirectional chargers and communication systems)^15^. However, subsidies and tax rebates are intended to close the price gap between traditional automobiles and EVs.

The influence of economic factors (such as governmental subsidies, manufacturers’ cash incentives, total cost of ownership (TCO), fuel costs and battery prices) on EV customers should be carefully addressed and analysed for EV economic acceptance^160^. Compared with traditional EVs, which are noticeable in regions with high fossil fuel taxes and low electricity prices, such as Norway, EVs may offer potential cost reductions in operations and emissions^161^. Additionally, subsidies play a vital role in encouraging BEVs, which in turn boosts economic activity^160^. EV transition creates new business opportunities in the market, which can drive economic growth through new job creation^12^. Despite the long-term savings of EVs, their TCO plays an important role in consumer decision-making since the initial cost of EVs presents a significant barrier to their adoption^161^, especially in countries and governments with fewer subsidies and incentive policies. Thus, increasing research and studies to simplify the implementation of V2G technology, reduce its initial and running costs, decrease the cost of EV batteries and their degradation time, and achieve the development of an EV charging infrastructure are important. In conclusion, the complex relationships among economic aspects, governmental policies, consumer behaviour, and market dynamics strongly shape the adoption of EVs.

#### V2G commercialization

EV-owner-based studies reveal that there are other potential barriers to V2G adoption, the most important of which are consumer unawareness of grid integration benefits and concerns about V2G programs in terms of privacy, cost and loss of control^36^. Thus, to improve V2G commercialization, a number of issues should be addressed^15^.

- Development of economic viability metrics and models for charging station infrastructure, which is particularly challenging in regions marked by fluctuating energy costs and evolving market dynamics
- Implementing new e-trading schemes and enabling the incorporation of wind and solar power as well as setting special tariff structures for the colocation of EVs and renewables to increase the revenue of EV owners from V2G.
- Promoting proper awareness among EV owners and providing sufficient benefits to promote V2G technology.

### Social aspects

Currently, communities are paying attention to the potential opportunities offered by EVs, especially with V2G technology, by encouraging the adoption of EVs by community residents and businesses and even transitioning community-owned vehicles to EVs. This would serve the community in many social aspects as follows:

Public health: Most school buses and public transportation run on diesel fuel, which emits harmful gases that affect brain development and contribute to asthma and cancer. Every school day, 20 million children are exposed to harmful fumes from school buses^162^; thus, electrification is particularly appealing with these types of vehicles to enhance community public health.

Fuel and operating cost savings: Another advantage to the community is the ability of EVs to meet budget constraints and challenges. Compared with gasoline or diesel fuel expenses, vehicle fuel expenses can be lowered by 50% or less because electricity costs per energy unit are lower at higher efficiency. Additionally, operating expenses such as those related to insurance, tires, registration, and maintenance are typically 25% lower than those of their conventional counterparts^163^

Job creation: The growth of V2G technology creates new job opportunities where the demand for skilled workers to install, maintain, and operate V2G-enabled charging stations increases.

However, some social challenges still exist that should be addressed, and some actions should be recommended and standardized when implementing V2G programs to encourage EV drivers to participate.

#### Social acceptance and perceptions of EVs

Despite the previously discussed benefits, critical areas that require deeper investigation to accelerate EV adoption and integration into society^2^. The high upfront costs of EVs and insufficient charging infrastructure may exclude lower-income and rural communities, leading to social inequalities^164^. Therefore, developing affordable EV models by manufacturers, adapting financial incentives and flexible governmental policies on the basis of income levels should be adapted worldwide to different economic backgrounds to reduce the cost of EV ownership, ensure equitable growth and promote EV adoption. Other factors influencing the public acceptance and adoption rates of EVs include those related to changes in transportation habits, concerns about charging availability, and the range of anxiety. Thus, developing a robust network of charging stations is essential to alleviate range anxiety and accelerate transport electrification^2^.

#### Awareness and knowledge of the benefits of EVs

Awareness of the benefits of EVs is an essential factor for increasing their market growth. Thus, training, educational programs and public campaigns are effective in increasing consumers’ familiarity with EV technology, increasing their social acceptance, removing any barriers to EV adoption and enabling the transition to a sustainable mobility and transportation system^2,22^. Moreover, customers who respect and value environmental attitudes and ecological sustainability are more likely to consider it a more environmentally friendly alternative. Therefore, awareness campaigns can increase this goal by highlighting the environmental benefits of EVs.

#### Marketing and advertising

Marketing and advertising could help shape stakeholders’ perceptions of the importance of financial investments in the EV context and V2G technology and stimulate consumers in its adoption. This implies the use of communication strategies, marketing organizations and advertising campaigns to generate new businesses for EV products, services and value^22^.

### Environmental aspect

Electric and plug-in hybrid vehicles are paving the way toward transportation decarbonization since they are quieter, cleaner, and more efficient than gasoline- and diesel-powered vehicles are^165^. Lacking tailpipes, EVs can directly eliminate harmful pollutant emissions, including particulates, ozone, carbon monoxide, and nitrogen oxides. Hence, in the transportation sector, replacing diesel trucks, buses, vans and passenger vehicles with EVs presents a promising opportunity for reducing pollutant exposure in communities and, in turn, enhancing public health. Although EVs present a promising solution for reducing greenhouse gas emissions and combating climate change, their adoption faces several environmental challenges, and related recommendations are suggested in Table 16 as follows:

#### Promotion of sustainable and clean transportation

In addition to being a sustainable, environmentally friendly alternative, the replacement of fossil fuel vehicles with EVs will shift the demand from crude oil to electricity and batteries. This can enhance energy security by lessening reliance on fossil fuel, which in turn, can increase in the stability of energy prices^166^. The latter can be an appealing factor to grab consumers’ attention towards this clean option. Another influencing factor on consumers’ intention to use electric vehicles is that greater EV penetration can help directly reduce the costs related to health problems associated with air pollution caused by fuel-based vehicles^167^. Hence, this will serve well for sustainable development goals, sdg3: good health and well-being and sdg7: affordable and clean energy^3^.

Accordingly, governments, especially in uncultured communities, are encouraged to promote environmentally friendly mobility through awareness campaigns and consumer education on the previously discussed environmental benefits of EVs and to develop strategies to encourage consumers’ social responsibility and green behaviour^2^. Moreover, governments are advised to increase the fuel oil tax and implement taxes on high-emission vehicles while offering other incentives for low-emission EVs to promote their relative competitiveness^168^.

#### EVs’ battery production and disposal

Despite the environmental benefits offered by EVs and V2G technology, with high penetration of EVs, the demand for batteries is increasing, thus environmental concerns can arise and should be studied^15^. This can be related to the process of batteries production and disposal. Regarding the widely used lithium-ion batteries, Li extraction and separation involves a lot of chemical procedures that are dangerous to the environment and can lead to health hazards. Moreover, materials used in EV batteries are challenging to recycle and careless disposal of these batteries can be toxic and may even lead to fire hazards^13^.

Hence, it is essential to have a well-defined, environment-friendly end-of-life (EOL) strategy for the batteries along with considering second-life batteries reusing which can contribute to better energy security^169^. Moreover, developments in the production of organic Li-batteries, along with safe repurposing and recycling of Li-batteries can make EV-technology greener, cleaner and more robust, affordable, and safe. Future research should focus on technical aspects and long-term impacts of battery composition, disposal and recycling to determine the overall environmental impact of EVs^169^.

#### Divided opinions

One major concern is the pollution generated in battery production, coupled with the insufficient infrastructural capacity for recycling used batteries, leading to divided opinions on the environmental merits of EVs^2^. Despite the potential for EVs to reduce CO2 emissions^168^, the environmental benefits are often doubted by consumers, who are uncertain about EV battery capacity^2^. Additionally, the high battery cost and the need to make EVs affordable for price-sensitive markets further complicate the scenario^170^. Since consumers’ concerns significantly influence their intention to adopt EVs, governments and industry manufacturers must allocate research resources for battery technology development and charging infrastructure expansion to help alleviate some customers’ concerns about EV adoption^167^.

## Discussion

Widespread penetration of EVs into the utility grid potentially impacts the power grid; thus, smart controlled charging is crucial in EV electrification to minimize the impact of uncontrolled charging on the power system. V2G technology enables bidirectional energy flow between EVs and the power grid, thus transforming EVs into valuable energy storage resources and unlocking numerous advantages. However, several future challenges remain to expand the use of this technology.

**Fig. 14:**
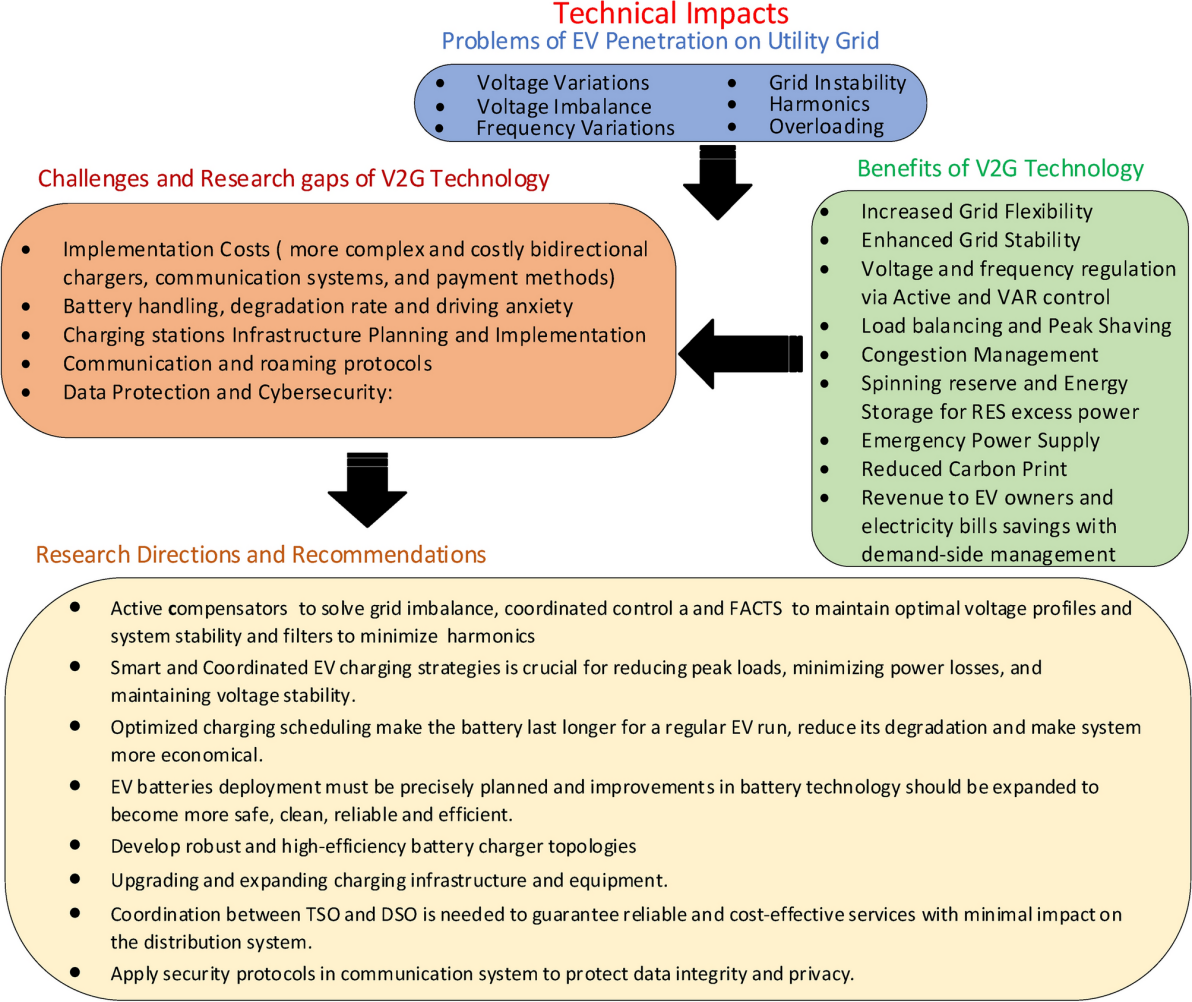
Technical impacts, challenges and research directions.

**Fig. 15:**
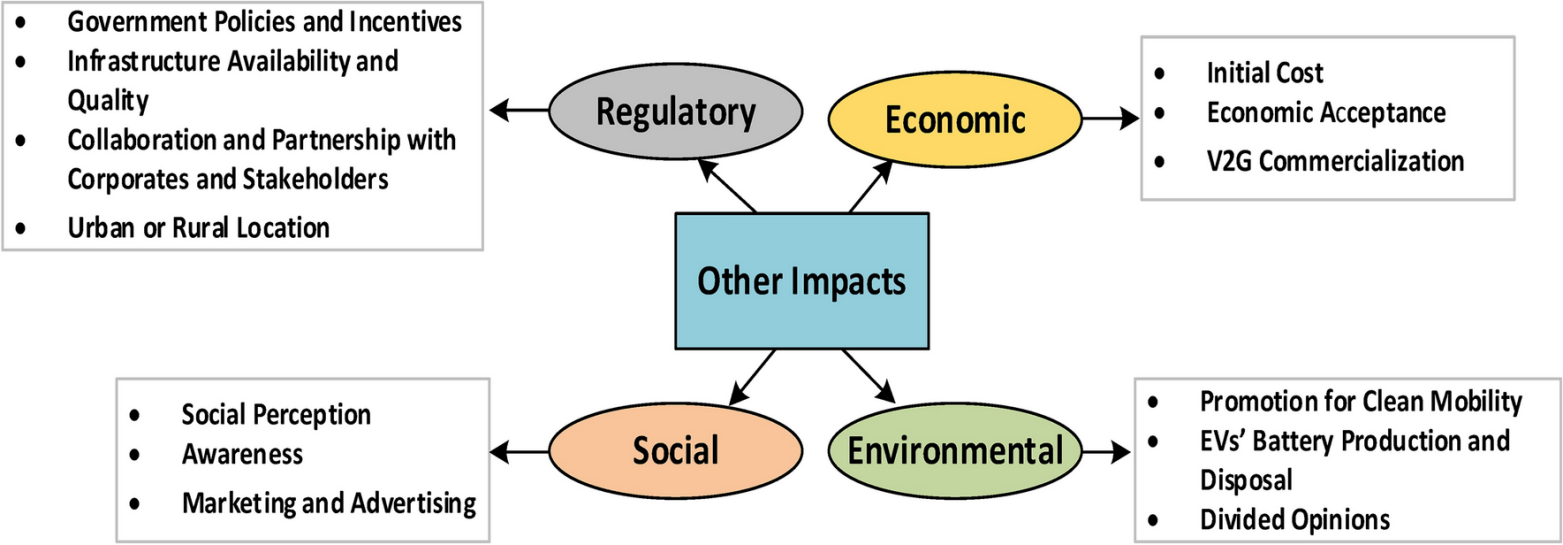
Aspects of other impacts.

In this paper, EV penetration problems on grids as well as the main benefits of V2G technology, related challenges and recommended research directions and remedies are discussed in detail and summarized in Fig. 14. Moreover, other critical factors, which are not widely investigated, are studied in this paper. This includes factors that are influential at the regulatory, social, economic and environmental levels. For all these factors, the main key findings, challenges and recommended measures are discussed, and the main issues covered by each of these impacts are highlighted in Fig. 15.

With respect to the prioritization of the multidimensional impacts associated with EV penetration, the technical concerns, specifically those affecting the power grid due to uncontrolled EV charging, will likely act first. This is related to the fact that the following technical issues are encountered^171^:

- Immediate Impact: As EV adoption increases, the most immediate and measurable impact, particularly in urban areas with high EV con concentrations, is the stress and overload imposed on the grid, especially during peak hours. This can be associated with voltage variations and imbalance along with harmonics and power quality issues, leading to potential outages or inefficiencies. The influence of each technical problem differs according to the penetration level, system ratings, RES integration and existing charging infrastructure capabilities.
- Mitigation Needs: Addressing the latter requires near-term investments in smart charging infrastructure, grid upgrades to balance loads effectively and mitigation techniques to attenuate these technical problems. Nevertheless, the cost and scalability of these mitigation techniques differ according to the nature of the adopted technique (hardware or software), where software solutions add to system control complexity, whereas hardware solutions add to system cost and size. The latter is also affected by the hardware solution topology, whether it includes high-cost power-electronic devices, featuring enhanced mitigation results, or relatively less cost passive elements yet with limited performance.

Notably, delays in addressing technical issues directly impact social acceptance, and economic incentives for wider adoption and even environmental benefits may not be fully realized. Hence, immediate resolution of technical challenges is mandatory, setting the stage for broader cross-linked aspects, including economic, social, and environmental transformations, as EV infrastructure matures as well as policies evolving to adapt to the new landscape.

With respect to the scalability of the multidimensional effects associated with EV penetration; varying scales of impacts are encountered^172^. Local, regional and national areas will initially face technical, social and economic challenges, whereas global efforts will extend to regulatory and environmental aspects. Table 17 summarizes the main enhancements required for each aspect, related effort costs and the corresponding scaling level.

**Table 17:** Scalability of each aspect.

Aspect	Enhancements	Efforts’ costs	Scale
Technical	Grid stability, infrastructure growth	Grid upgrades, smart charging, V2G infrastructure implementation	Local to National
Social	Behavioral change, EV /V2G acceptance	Public education, awareness campaigns, incentives, consumer outreach	Local to Regional
Economic	Market transformation, job shifts	New energy markets, job retraining, tax incentives and subsidies	Regional to National
Regulatory	Governmental Policies and incentives	Regulatory standards and subsidies as well as collaboration with corporates	Regional to Global
Environmental	Emissions reduction, battery waste disposal	Green energy investment, battery recycling	Local to Global

- Technical aspect: local to national scale

Immediate solutions will involve upgrading local grid infrastructure, smart meters, and establishing charging stations whereas long-term V2G investments involve national governments, regulatory bodies, and the private sector as they will require bi-directional charging technology, communication systems between vehicles and the grid, and regulatory frameworks for energy storage and distribution.

- Social aspect: local to regional scale

Investments in public awareness campaigns, incentives for EV adoption, and educational programs will be required at local and regional levels.

- Economic aspect: regional to national scale

The electricity market must witness transformation due to the need to adapt to new players (EV owners, aggregators) participating in energy generation and storage. This will include investments in setting up regional energy markets along with setting regulatory standards. Moreover, national governments may need to establish tax incentives, subsidies, and support programs to smooth the transition to EVs and V2G systems, especially for those economically displaced by the shift.

- Regulatory aspect: regional to global scale

National governments should set regulatory standards for regional energy generation, storage and charging scheduling as well as establish policies and incentives (tax breaks, subsidies) besides collaboration with private sectors to promote widespread EV use. Additionally, later on, international cooperation on standards and regulations will be necessary, requiring governmental negotiations and policy changes.

- Environmental aspects: local to global scale

Investments in greener electricity sources (e.g., wind, solar) at the local and regional levels will drive the environmental benefit but require significant initial capital. International grants and partnerships with private companies will be essential. To mitigate battery waste, large-scale recycling facilities and regulations as well as global research on battery disposal will be required.

**Fig. 16:**
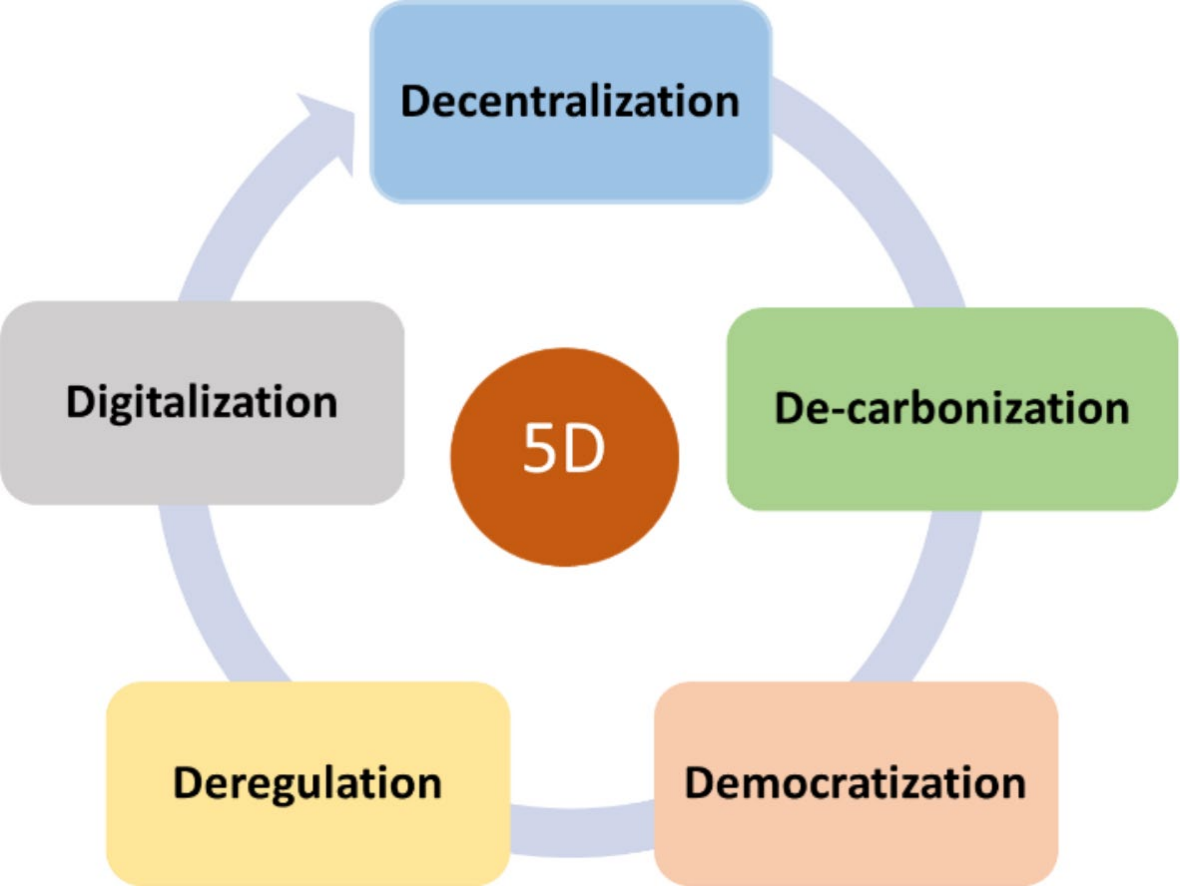
5D vision of V2G technology in smart grid systems^19^.

Finally, for a broad integrated outlook on all the previously discussed factors, the 5D vision is a promising tool for designing and implementing V2G technology. The 5D vision, as shown in Fig. 16, is a newly developed outlook offering different perspectives when dealing with issues and policies arising in EVs in the future and covering overall EV applications to satisfy multiple stakeholders^19^. The main advantage of 5D vision is that it provides a digital platform for future EV implementations, thus maintaining the following sustainable development goals:

- Decentralization: Various noncentralized control schemes that address a wide range of input and output parameters and exchange data between EVs, aggregators and control centers throughout the utility grid.
- Decarbonization: Supporting a clean green environment for both the utility grid and transportation system.
- Democratization: Distributed generation via diverse independent power producers supporting V2X technology.
- Deregulation: Regulating energy sales and deciding from where to buy convenient demand on the basis of the electricity market for a win‒win game to the utility and EV owners.
- Digitalization: Transformation of EV generations and development of new ones using digital technology.

## Conclusion

Owing to its environmental, social and economic benefits compared to traditional vehicles, there is a persistent need to expanding EVs adoption and integration to utility grid. However, uncoordinated charging results in negative impacts imposed on the utility upon which V2G technology is developed offering enhanced grid stability, increased integration of renewable energy, load balancing and financial incentives for EV owners. This paper presents different EVs technologies as well as V2G infrastructure. This is followed by a detailed quantified analysis of the technical problems reflected on the utility, in case of uncontrolled EV charging, along with the corresponding solutions. In addition, the current need for the promising V2G technology and its benefits in achieving a more stable, reliable and eco-friendly utility grid are discussed. Besides these technical aspects, the proposed study covers other critical factors that influence V2G adoption politically, environmentally, socially and economically. Moreover, this study contributes in highlighting challenges and knowledge gaps facing each of the studied impacts, then driving respective recommendations and research directions to enhance the adoption and social acceptance of this technology.

Hence, this study provides useful information and suggest measures that can guide decision-makers to establish required policies and manufacturers to develop standards and advancements towards EV charging scheduling, coordination and infrastructure upgrading and expanding. Moreover, this will encourage governments to offer subsidies to EV manufacturers and incentives to EV consumers as well as establishing partnerships with third parties and holding awareness campaigns to increase the acceptance and accelerate adoption of V2G technology among community. Finally, this will also direct research towards new trends of safe battery production, handling and disposal as well as secure communication and 5Ds Vision scheme. In conclusion, the proposed study is targeted at hastening the transition to V2G by identifying benefits, influencing factors and challenges, emphasizing research patterns and drawing recommendations and tangible conclusions.

## Data Availability

All data used to support the findings of this study are included in the article.

## References

[1] IEA. (2023). Global EV Outlook 2023 (2023).

[2] Pamidimukkala, A., Kermanshachi, S., Rosenberger, J. M. & Hladik, G. Evaluation of barriers to electric vehicle adoption: A study of technological, environmental, financial, and infrastructure factors. *Transp. Res. Interdiscip. Perspect.* **22**, 100962. 10.1016/j.trip.2023.100962 (2023).

[3] D. of E. and S. A. S. D. United Nations, “THE 17 GOALS”. https://sdgs.un.org/goals. Accessed 14 Jul 2024.

[4] Sanguesa, J. A., Torres-Sanz, V., Garrido, P., Martinez, F. J. & Marquez-Barja, J. M. A review on electric vehicles: Technologies and challenges. *Smart Cities* **4**(1), 372–404. 10.3390/smartcities4010022 (2021).

[5] Rivera, S. et al. Electric vehicle charging infrastructure: From grid to battery. *IEEE Ind. Electron. Mag.* **15**(2), 37–51. 10.1109/MIE.2020.3039039 (2021).

[6] Habib, S. et al. A comprehensive study of implemented international standards, technical challenges, impacts and prospects for electric vehicles. *IEEE Access* **6**, 13866–13890. 10.1109/ACCESS.2018.2812303 (2018).

[7] Berkeley, N., Jarvis, D. & Jones, A. Analysing the take up of battery electric vehicles: An investigation of barriers amongst drivers in the UK. *Transp. Res. D Transp. Environ.* **63**, 466–481. 10.1016/j.trd.2018.06.016 (2018).

[8] Li, X. et al. Electric vehicle behavior modeling and applications in vehicle-grid integration: An overview. *Energy* **268**, 126647. 10.1016/j.energy.2023.126647 (2023).

[9] Alshahrani, S., Khalid, M. & Almuhaini, M. Electric vehicles beyond energy storage and modern power networks: Challenges and applications. *IEEE Access* **7**, 99031–99064. 10.1109/ACCESS.2019.2928639 (2019).

[10] Yilmaz, M. & Krein, P. T. Review of the impact of vehicle-to-grid technologies on distribution systems and utility interfaces. *IEEE Trans. Power Electron.* **28**(12), 5673–5689. 10.1109/TPEL.2012.2227500 (2013).

[11] Pani, P., Athreya, A. R., Panday, A., Bansal, H. O. & Agrawal, H. P. Integration of the vehicle-to-grid technology. in *2015 International Conference on Energy Economics and Environment (ICEEE)* 1–5 (IEEE, 2015). 10.1109/EnergyEconomics.2015.7235108.

[12] Mojumder, M. R. H., Ahmed Antara, F., Hasanuzzaman, M., Alamri, B. & Alsharef, M. Electric vehicle-to-grid (V2G) technologies: Impact on the power grid and battery. *Sustainability*10.3390/su142113856 (2022).

[13] Hossain, M. S., Kumar, L., El Haj Assad, M. & Alayi, R. *Advancements and Future Prospects of Electric Vehicle Technologies: A Comprehensive Review* (Hindawi Limited, 2022). 10.1155/2022/3304796.

[14] Gnanavendan, S. et al. Challenges, solutions and future trends in EV-technology: A review. *IEEE Access* **12**, 17242–17260. 10.1109/ACCESS.2024.3353378 (2024).

[15] Vishnu, G. et al. *Review of Challenges and Opportunities in the Integration of Electric Vehicles to the Grid* (Multidisciplinary Digital Publishing Institute (MDPI), 2023). 10.3390/wevj14090259.

[16] Mahmud, I., Medha, M. B. & Hasanuzzaman, M. Global challenges of electric vehicle charging systems and its future prospects: A review. *Res. Transp. Bus. Manag.*10.1016/j.rtbm.2023.101011 (2023).

[17] Tirunagari, S., Gu, M. & Meegahapola, L. Reaping the Benefits of Smart Electric Vehicle Charging and Vehicle-to-Grid Technologies: Regulatory, Policy and Technical Aspects. *IEEE Access* **10**, 114657–114672. 10.1109/ACCESS.2022.3217525 (2022).

[18] Nour, M., Chaves-Ávila, J. P., Magdy, G. & Sánchez-Miralles, Á. *Review of Positive and Negative Impacts of Electric Vehicles Charging on Electric Power Systems* (MDPI AG, 2020). 10.3390/en13184675.

[19] Ismail, A. A. et al. Impact of electric vehicles on smart grid and future predictions: A survey. *Int. J. Model. Simulat.* **43**(6), 1041–1057. 10.1080/02286203.2022.2148180 (2023).

[20] Nutkani, I., Toole, H., Fernando, N. & Andrew, L. P. C. *Impact of EV charging on electrical distribution network and mitigating solutions: A review* (Wiley, 2024). 10.1049/stg2.12156.

[21] Hossain, S. et al. Grid-vehicle-grid (G2V2G) Efficient power transmission: An overview of concept, operations, benefits, concerns, and future challenges. *Sustainability* **15**(7), 5782. 10.3390/su15075782 (2023).

[22] Singh, V., Singh, H., Dhiman, B., Kumar, N. & Singh, T. Analyzing bibliometric and thematic patterns in the transition to sustainable transportation: Uncovering the influences on electric vehicle adoption. *Res. Transp. Bus. Manag.* **50**, 101033. 10.1016/j.rtbm.2023.101033 (2023).

[23] Shariff, S. M. et al. A state-of-the-art review on the impact of fast EV charging on the utility sector. *Energy Stor.*10.1002/est2.300 (2022).

[24] Acharige, S. S. G. et al. Review of electric vehicle charging technologies, standards, architectures, and converter configurations. *IEEE Access* **11**, 41218–41255. 10.1109/ACCESS.2023.3267164 (2023).

[25] Aghajan-Eshkevari, S., Azad, S., Nazari-Heris, M., Ameli, M. T. & Asadi, S. Charging and discharging of electric vehicles in power systems: An updated and detailed review of methods, control structures, objectives, and optimization methodologies. *Sustainability* **14**(4), 2137. 10.3390/su14042137 (2022).

[26] Ibrahim, R. A. & Gaber, I. M. Electric vehicles: From charging infrastructure to impacts on utility grid. in *Advances in Transdisciplinary Engineering* 326–337 (IOS Press BV, 2024). 10.3233/ATDE240332

[27] Grenier, M., Aghdam, M. G. H. & Thiringer, T. Design of on-board charger for plug-in hybrid electric vehicle. *IET Conf. Publ.*10.1049/cp.2010.0101 (2010).

[28] Haghbin, S. *et al.*, Integrated chargers for EV’s and PHEV’s: Examples and new solutions. in *19th International Conference on Electrical Machines* (*ICEM 2010*, 2010). 10.1109/ICELMACH.2010.5608152

[29] International Electrotechnical Commission. and Commission electrotechnique internationale., *Electric vehicle conductive charging system. Part 1, General requirements = Systéme de charge conductive pour véhicules électriques. Partie 1, Exigences générales* (International Electrotechnical Commission, 2017).

[30] Hybrid - EV Committee. SAE Electric Vehicle and Plug in Hybrid Electric Vehicle Conductive Charge Coupler J1772_201710. SAE International. https://www.sae.org/standards/content/j1772_201710/. Accessed 17 Mar 2024.

[31] Botsford, C. & Szczepanek, A. Fast charging versus slow charging: Pros and cons for the new age of electric vehicles. in *24th International Battery, Hybrid and Fuel Cell Electric Vehicle Symposium and Exhibition 2009, EVS 24* (2009).

[32] Chen, T. et al. A review on electric vehicle charging infrastructure development in the UK. *J. Mod. Power Syst. Clean Energy* **8**(2), 193–205. 10.35833/MPCE.2018.000374 (2020).

[33] Kempton, W. & Tomić, J. Vehicle-to-grid power fundamentals: Calculating capacity and net revenue. *J. Power Sources*10.1016/j.jpowsour.2004.12.025 (2005).

[34] Yilmaz, M. & Krein, P. T. Review of the impact of vehicle-to-grid technologies on distribution systems and utility interfaces. *IEEE Trans. Power Electron.*10.1109/TPEL.2012.2227500 (2013).

[35] Kempton, W. & Tomić, J. Vehicle-to-grid power implementation: From stabilizing the grid to supporting large-scale renewable energy. *J. Power Sources*10.1016/j.jpowsour.2004.12.022 (2005).

[36] Mastoi, M. S. et al. *A Study of Charging-Dispatch Strategies and Vehicle-to-Grid Technologies for Electric Vehicles in Distribution Networks* (Elsevier Ltd, 2023). 10.1016/j.egyr.2022.12.139.

[37] Escudero-Garzas, J. J., Garcia-Armada, A. & Seco-Granados, G. Fair design of plug-in electric vehicles aggregator for V2G regulation. *IEEE Trans. Veh. Technol.*10.1109/TVT.2012.2212218 (2012).

[38] Rajasekaran, A. S., Azees, M. & Al-Turjman, F. A comprehensive survey on security issues in vehicle-to-grid networks. *J. Control Decis.*10.1080/23307706.2021.2021113 (2023).

[39] Oad, A., Ahmad, H. G., Talpur, M. S. H., Zhao, C. & Pervez, A. Green smart grid predictive analysis to integrate sustainable energy of emerging V2G in smart city technologies. *Optik (Stuttg)*10.1016/j.ijleo.2022.170146 (2023).

[40] Monteiro, V., Pinto, J. G. & Afonso, J. L. Operation modes for the electric vehicle in smart grids and smart homes: Present and proposed modes. *IEEE Trans. Veh. Technol.*10.1109/TVT.2015.2481005 (2016).

[41] M. S, A. A & Vasudha, K. Power Flow in hybrid electric vehicles and battery electric vehicles. in *EAI/Springer Innovations in Communication and Computing*, 2022. 10.1007/978-3-030-85424-9_6.

[42] Ahmadian, A., Mohammadi-Ivatloo, B. & Elkamel, A. A review on plug-in electric vehicles: Introduction, current status, and load modeling techniques. *J. Mod. Power Syst. Clean Energy* **8**(3), 412–425. 10.35833/MPCE.2018.000802 (2020).

[43] Ibrahim, R. A., Hamad, M. S., Dessouky, Y. G. & Williams, B. W. A novel topology for enhancing the low voltage ride through capability for grid connected wind turbine generators. in *2012 IEEE Energy Conversion Congress and Exposition (ECCE)* 2389–2395 (IEEE, 2012). 10.1109/ECCE.2012.6342456

[44] Ibrahim, R. A., Ahmed, K. H., Williams, B. W., Hamad, M. S. & Dessouky, Y. G. Improved ride-through of PMSG wind turbine during symmetrical voltage dip using a magnetic amplifier. in *2nd IET Renewable Power Generation Conference (RPG 2013)* 3.51–3.51 (Institution of Engineering and Technology, 2013). 10.1049/cp.2013.1857

[45] Lee, S. J. et al. Evaluation of voltage sag and unbalance due to the system connection of electric vehicles on distribution system. *J. Electr. Eng. Technol.*10.5370/JEET.2014.9.2.452 (2014).

[46] Singh, S. & Singh, S. P. Peak load relief in MV/LV distribution networks through smart grid-enabled CVR with droop control EV2G reactive power support. in *2018 International Conference on Power, Instrumentation, Control and Computing (PICC)* 1–6 (IEEE, 2018). 10.1109/PICC.2018.8384794

[47] Khan, S. U. et al. Energy management scheme for an EV smart charger V2G/G2V application with an EV power allocation technique and voltage regulation. *Appl. Sci. (Switz.)*10.3390/app8040648 (2018).

[48] Saadeh, O., Al Nawasrah, A. & Dalala, Z. A bidirectional electrical vehicle charger and grid interface for grid voltage dip mitigation. *Energies (Basel)*10.3390/en13153784 (2020).

[49] Rahman Aliffianto, L., Suyono, H. & Soekotjo Dachlan, H. Coordination of charging plug-in electric vehicles (PEV) in electric distribution networks to minimize power losses and voltage deviations. www.ijcat.com

[50] Adewuyi, O. B., Shigenobu, R., Ooya, K., Senjyu, T. & Howlader, A. M. Static voltage stability improvement with battery energy storage considering optimal control of active and reactive power injection. *Electr. Power Syst. Res.* **172**, 303–312. 10.1016/j.epsr.2019.04.004 (2019).

[51] Çelik, D. & Meral, M. E. A coordinated virtual impedance control scheme for three phase four leg inverters of electric vehicle to grid (V2G). *Energy*10.1016/j.energy.2022.123354 (2022).

[52] Liu, M., Phanivong, P. K., Shi, Y. & Callaway, D. S. Decentralized charging control of electric vehicles in residential distribution networks. *IEEE Trans. Control Syst. Technol.* **27**(1), 266–281. 10.1109/TCST.2017.2771307 (2019).

[53] Cao, C., Wu, Z. & Chen, B. Electric vehicle-grid integration with voltage regulation in radial distribution networks. *Energies (Basel)*10.3390/en13071802 (2020).

[54] Fang, X., Li, J., Yuan, Y. & Yuan, X. Decentralized control strategy for participation of electric vehicles in distribution voltage control. *Processes*10.3390/pr11092552 (2023).

[55] Kriukov, A. et al. Novel decentralized voltage-centered EV charging control algorithm using DSRC system in low voltage distribution networks. *IEEE Access* **9**, 164779–164800. 10.1109/ACCESS.2021.3132419 (2021).

[56] Hu, J., Ye, C., Ding, Y., Tang, J. & Liu, S. A distributed MPC to exploit reactive power V2G for real-time voltage regulation in distribution networks. *IEEE Trans. Smart Grid* **13**(1), 576–588. 10.1109/TSG.2021.3109453 (2022).

[57] Xi, H. et al. Centralized and decentralized combined voltage control for distribution network with high penetration PV connected. *J. Phys. Conf. Ser.* **2771**(1), 012040. 10.1088/1742-6596/2771/1/012040 (2024).

[58] Zakzouk, N. E., Khamis, A. K., Abdelsalam, A. K. & Williams, B. W. Continuous-input continuous-output current buck-boost DC/DC converters for renewable energy applications: Modelling and performance assessment. *Energies (Basel)* **12**(11), 2208. 10.3390/en12112208 (2019).

[59] Islam, M. R., Lu, H., Hossain, M. J. & Li, L. Mitigating unbalance using distributed network reconfiguration techniques in distributed power generation grids with services for electric vehicles: A review. *J. Clean. Prod.* **239**, 117932. 10.1016/j.jclepro.2019.117932 (2019).

[60] Nájera, J., Mendonça, H., de Castro, R. & Arribas, J. Strategies comparison for voltage unbalance mitigation in LV distribution networks using EV chargers. *Electronics (Basel)* **8**(3), 289. 10.3390/electronics8030289 (2019).

[61] Pinthurat, W., Hredzak, B., Konstantinou, G. & Fletcher, J. Techniques for compensation of unbalanced conditions in LV distribution networks with integrated renewable generation: An overview. *Electr. Power Syst. Res.* **214**, 108932. 10.1016/j.epsr.2022.108932 (2023).

[62] Mishra, A., Tripathy, M. & Ray, P. A survey on different techniques for distribution network reconfiguration. *J. Eng. Res.*10.1016/j.jer.2023.09.001 (2023).

[63] Kongtrakul, N., Wangdee, W. & Chantaraskul, S. Comprehensive review and a novel technique on voltage unbalance compensation. *IET Smart Grid* **6**(4), 331–358. 10.1049/stg2.12106 (2023).

[64] de Araujo, L. R., Penido, D. R. R., Carneiro, S. & Pereira, J. L. R. Optimal unbalanced capacitor placement in distribution systems for voltage control and energy losses minimization. *Electr. Power Syst. Res.* **154**, 110–121. 10.1016/j.epsr.2017.08.012 (2018).

[65] Omar, R. & Rahim, N. A. Voltage unbalanced compensation using dynamic voltage restorer based on supercapacitor. *Int. J. Electr. Power Energy Syst.* **43**(1), 573–581. 10.1016/j.ijepes.2012.05.015 (2012).

[66] Nayeripour, M., Mahboubi-Moghaddam, E., Narimani, M. R. & Waffenschmidt, E. Secure and reliable distribution feeder reconfiguration in the presence of static VAR compensator. *Iran. J. Sci. Technol. Trans. Electr. Eng.* **44**(1), 293–308. 10.1007/s40998-019-00243-1 (2020).

[67] Zhang, Z., He, L. & Wu, S. Reconfiguration strategy of multi-objective optimal dispatching with distributed generation. in *2020 10th International Conference on Power and Energy Systems (ICPES)* 99–103 (IEEE, 2020). 10.1109/ICPES51309.2020.9349727

[68] AzamMuhammad, M. et al. Distribution network planning enhancement via network reconfiguration and DG integration using dataset approach and water cycle algorithm. *J. Mod. Power Syst. Clean Energy* **8**(1), 86–93. 10.35833/MPCE.2018.000503 (2020).

[69] Azizivahed, A. et al. Multi-objective dynamic distribution feeder reconfiguration in automated distribution systems. *Energy* **147**, 896–914. 10.1016/j.energy.2018.01.111 (2018).

[70] Salkuti, S. R. Optimal operation of microgrid considering renewable energy sources, electric vehicles and demand response. *E3S Web of Conf.* **87**, 01007. 10.1051/e3sconf/20198701007 (2019).

[71] Shanmugapriya, P., Baskaran, J., Nayanatara, C. & Kothari, D. P. IoT based approach in a power system network for optimizing distributed generation parameters. *Comput. Model. Eng. Sci.* **119**(3), 541–558. 10.32604/cmes.2019.04074 (2019).

[72] B. E. Sedhom, M. M. El-Saadawi, M. S. El Moursi, Mohamed. A. Hassan, and A. A. Eladl, “IoT-based optimal demand side management and control scheme for smart microgrid,” *International Journal of Electrical Power & Energy Systems*, vol. 127, p. 106674, May 2021, 10.1016/j.ijepes.2020.106674.

[73] Ahmed, H. M. A., Ahmed, M. H. & Salama, M. M. A. Network reconfiguration for the optimal operation of smart distribution systems. in *2019 IEEE Power & Energy Society General Meeting (PESGM)* 1–5 (IEEE, 2019). 10.1109/PESGM40551.2019.8973639

[74] Ahmed, H. M. A. & Salama, M. M. A. Energy management of AC–DC hybrid distribution systems considering network reconfiguration. *IEEE Trans. Power Syst.* **34**(6), 4583–4594. 10.1109/TPWRS.2019.2916227 (2019).

[75] Barman, P. et al. Renewable energy integration with electric vehicle technology: A review of the existing smart charging approaches. *Renew. Sustain. Energy Rev.* **183**, 113518. 10.1016/j.rser.2023.113518 (2023).

[76] Islam, Md. R., Lu, H., Hossain, M. J. & Li, L. Optimal coordination of electric vehicles and distributed generators for voltage unbalance and neutral current compensation. *IEEE Trans. Ind. Appl.* **57**(1), 1069–1080. 10.1109/TIA.2020.3037275 (2021).

[77] Then, J., Agalgaonkar, A. P. & Muttaqi, K. M. Coordinated charging of spatially distributed electric vehicles for mitigating voltage rise and voltage unbalance in modern distribution networks. *IEEE Trans. Ind. Appl.*10.1109/TIA.2023.3273186 (2023).

[78] Bozalakov, D. V., Mnati, M. J., Laveyne, J., Van den Bossche, A. & Vandevelde, L. Voltage unbalance and overvoltage mitigation by using the three-phase damping control strategy in battery storage applications. in *2018 7th International Conference on Renewable Energy Research and Applications (ICRERA)* 753–759 (IEEE, 2018). 10.1109/ICRERA.2018.8566779

[79] Wang, H., Yan, Z., Shahidehpour, M., Zhou, Q. & Xu, X. Optimal energy storage allocation for mitigating the unbalance in active distribution network via uncertainty quantification. *IEEE Trans. Sustain. Energy* **12**(1), 303–313. 10.1109/TSTE.2020.2992960 (2021).

[80] Gusev, Y. P. & Subbotin, P. V. Using battery energy storage systems for load balancing and reactive power compensation in distribution grids. in *2019 International Conference on Industrial Engineering, Applications and Manufacturing (ICIEAM)* 1–5 (IEEE, 2019). 10.1109/ICIEAM.2019.8742909

[81] Ranaweera, I., Midtgard, O.-M., Korpas, M. & Farahmand, H. Control strategies for residential battery energy storage systems coupled with PV systems. in *2017 IEEE International Conference on Environment and Electrical Engineering and 2017 IEEE Industrial and Commercial Power Systems Europe (EEEIC / I&CPS Europe)* 1–6 (IEEE, 2017). 10.1109/EEEIC.2017.7977466

[82] Nguyen, H. T. et al. Enhanced performance of charging stations via converter control under unbalanced and harmonic distorted grids. *IEEE Trans. Power Del.* **36**(6), 3964–3976. 10.1109/TPWRD.2021.3052319 (2021).

[83] Khalid, M. R., Khan, I. A., Hameed, S., Asghar, M. S. J. & Ro, J.-S. A Comprehensive review on structural topologies, power levels, energy storage systems, and standards for electric vehicle charging stations and their impacts on grid. *IEEE Access* **9**, 128069–128094. 10.1109/ACCESS.2021.3112189 (2021).

[84] Rimal, B., Kong, C., Poudel, B., Wang, Y. & Shahi, P. Smart electric vehicle charging in the era of internet of vehicles, emerging trends, and open issues. *Energies (Basel)* **15**(5), 1908. 10.3390/en15051908 (2022).

[85] Momoh, K. et al. State-of-the-art grid stability improvement techniques for electric vehicle fast-charging stations for future outlooks. *Energies (Basel)* **16**(9), 3956. 10.3390/en16093956 (2023).

[86] Adetokun, B. B. & Muriithi, C. M. Application and control of flexible alternating current transmission system devices for voltage stability enhancement of renewable-integrated power grid: A comprehensive review. *Heliyon* **7**(3), e06461. 10.1016/j.heliyon.2021.e06461 (2021). 33748502 10.1016/j.heliyon.2021.e06461 PMC7966839

[87] Saadaoui, A., Ouassaid, M. & Maaroufi, M. Overview of Integration of power electronic topologies and advanced control techniques of ultra-fast EV charging stations in standalone microgrids. *Energies (Basel)* **16**(3), 1031. 10.3390/en16031031 (2023).

[88] Roldan-Perez, J., Rodriguez-Cabero, A. & Prodanovic, M. Harmonic virtual impedance design for a synchronverter-based battery interface converter. in *2017 IEEE 6th International Conference on Renewable Energy Research and Applications (ICRERA)* 774–779 (IEEE, 2017). 10.1109/ICRERA.2017.8191164

[89] Muftau, B. & Fazeli, M. The role of virtual synchronous machines in future power systems: A review and future trends. *Electr. Power Syst. Res.* **206**, 107775. 10.1016/j.epsr.2022.107775 (2022).

[90] Errahimi, F., Essbai, N., ElIdrissi, Z. & Cheddadi, Y. Robust integral sliding mode controller design of a bidirectional DC charger in PV-EV charging station. *Int. J. Digit. Sign. Smart Syst.* **5**(2), 137. 10.1504/IJDSSS.2021.10036181 (2021).

[91] Shahjalal, M. et al. A critical review on charging technologies of electric vehicles. *Energies (Basel)* **15**(21), 8239. 10.3390/en15218239 (2022).

[92] Thangavel, S. et al. A comprehensive review on electric vehicle: Battery management system, charging station, traction motors. *IEEE Access* **11**, 20994–21019. 10.1109/ACCESS.2023.3250221 (2023).

[93] Hoffmann, F. et al. A multiport partial power processing converter with energy storage integration for EV stationary charging. *IEEE J. Emerg. Sel. Top Power Electron.* **10**(6), 7950–7962. 10.1109/JESTPE.2021.3102180 (2022).

[94] Sang, W. et al. Virtual synchronous generator, a comprehensive overview. *Energies (Basel)* **15**(17), 6148. 10.3390/en15176148 (2022).

[95] Singh, B. & Yadav, M. K. GA for enhancement of system performance by DG incorporated with D-STATCOM in distribution power networks. *J. Electr. Syst. Inf. Technol.* **5**(3), 388–426. 10.1016/j.jesit.2018.02.005 (2018).

[96] Zhou, X. et al. Control strategy research of D-STATCOM using active disturbance rejection control based on total disturbance error compensation. *IEEE Access* **9**, 50138–50150. 10.1109/ACCESS.2021.3069293 (2021).

[97] Shafiullah, M., Rana, M. J., Shahriar, M. S. & Zahir, M. H. Low-frequency oscillation damping in the electric network through the optimal design of UPFC coordinated PSS employing MGGP. *Measurement* **138**, 118–131. 10.1016/j.measurement.2019.02.026 (2019).

[98] Paital, S. R., Ray, P. K. & Mohanty, S. R. A robust dual interval type-2 fuzzy lead–lag based UPFC for stability enhancement using Harris Hawks optimization. *ISA Trans* **123**, 425–442. 10.1016/j.isatra.2021.05.029 (2022). 34119306 10.1016/j.isatra.2021.05.029

[99] Rashad, A., Kamel, S. & Jurado, F. Stability improvement of power systems connected with developed wind farms using SSSC controller. *Ain Shams Eng. J.* **9**(4), 2767–2779. 10.1016/j.asej.2017.03.015 (2018).

[100] Sahu, P. R., Hota, P. K. & Panda, S. Power system stability enhancement by fractional order multi input SSSC based controller employing whale optimization algorithm. *J. Electr. Syst. Inf. Technol.* **5**(3), 326–336. 10.1016/j.jesit.2018.02.008 (2018).

[101] Gasperic, S. & Mihalic, R. Estimation of the efficiency of FACTS devices for voltage-stability enhancement with PV area criteria. *Renew. Sustain. Energy Rev.* **105**, 144–156. 10.1016/j.rser.2019.01.039 (2019).

[102] Topel, M. & Grundius, J. Load management strategies to increase electric vehicle penetration-case study on a local distribution network in stockholm. *Energies (Basel)*10.3390/en13184809 (2020).

[103] Jenn, A. & Highleyman, J. Distribution grid impacts of electric vehicles: A California case study. *iScience*10.1016/j.isci.2021.103686 (2022). 35036872 10.1016/j.isci.2021.103686 PMC8749456

[104] Roy, P. et al. Impact of electric vehicle charging on power distribution systems: A case study of the grid in western kentucky. *IEEE Access* **11**, 49002–49023. 10.1109/ACCESS.2023.3276928 (2023).

[105] Crozier, C., Morstyn, T. & McCulloch, M. The opportunity for smart charging to mitigate the impact of electric vehicles on transmission and distribution systems. *Appl. Energy*10.1016/j.apenergy.2020.114973 (2020).

[106] Afshar, S., Pecenak, Z. K., Barati, M. & Disfani, V. Mobile charging stations for EV charging management in urban areas: A case study in Chattanooga. *Appl. Energy*10.1016/j.apenergy.2022.119901 (2022).

[107] S. M. S. A. A. & Morsy Nour, C. F. Smart Charging of electric vehicles according to electricity price. in *International Conference on Innovative Trends in Computer Engineering (ITCE’2019)* (Aswan: IEEE, 2019).

[108] Suski, A. et al. Analyzing electric vehicle load impact on power systems: Modeling analysis and a case study for Maldives. *IEEE Access* **9**, 125640–125657. 10.1109/ACCESS.2021.3111001 (2021).

[109] de Bitencourt, L. A. et al. Electric vehicles charging optimization to improve the insertion considering the brazilian regulatory framework. *Energy Storage*10.1002/est2.76 (2019).

[110] Lauvergne, R., Perez, Y., Françon, M. & Tejeda De La Cruz, A. Integration of electric vehicles into transmission grids: A case study on generation adequacy in Europe in 2040. *Appl. Energy*10.1016/j.apenergy.2022.120030 (2022).

[111] Basmadjian, R. Communication vulnerabilities in electric mobility hcp systems: A semi-quantitative analysis. *Smart Cities* **4**(1), 405–428. 10.3390/smartcities4010023 (2021).

[112] Misra, R., Paudyal, S., Ceylan, O. & Mandal, P. Harmonic distortion minimization in power grids withwind and electric vehicles. *Energies (Basel)*10.3390/en10070932 (2017).

[113] Pankaj, S., Khalid, M. R., Saad Alam, M., Jamil Asghar, M. S. & Hameed, S. Electric vehicle charging stations and their impact on the power quality of utility grid, in *2022 International Conference on Decision Aid Sciences and Applications (DASA)* 816–821 (IEEE, 2022). 10.1109/DASA54658.2022.9765054

[114] Song, T. et al. Suppression method of current harmonic for three-phase PWM rectifier in EV charging system. *IEEE Trans. Veh. Technol.* **69**(9), 9634–9642. 10.1109/TVT.2020.3005173 (2020).

[115] Jafari Siahroodi, H., Mojallali, H. & Mohtavipour, S. S. A new optimization framework for harmonic compensation considering plug-in electric vehicle penetration using adaptive particularly tunable fuzzy chaotic particle swarm optimization. *Energy Technol.*10.1002/ente.202000564 (2021).

[116] Nguyen, V.-L., Tran-Quoc, T. & Bacha, S. harmonic distortion mitigation for electric vehicle fast charging systems.

[117] Alame, D., Azzouz, M. & Kar, N. Assessing and mitigating impacts of electric vehicle harmonic currents on distribution systems. *Energies (Basel)*10.3390/en13123257 (2020).

[118] Çelik, D. Lyapunov based harmonic compensation and charging with three phase shunt active power filter in electrical vehicle applications. *Int. J. Electr. Power Energy Syst.*10.1016/j.ijepes.2021.107564 (2022).

[119] Hossain, S. et al. Grid-vehicle-grid (G2V2G) efficient power transmission: An overview of concept, operations, benefits, concerns, and future challenges. *Sustainability*10.3390/su15075782 (2023).

[120] Ghaebi Panah, P., Hooshmand, R. A., Gholipour, M. & Bornapour, M. Urban microgrid ancillary service provision using plugin electric vehicle and waste-to-energy CHP. *J. Energy Stor.*10.1016/j.est.2020.101413 (2020).

[121] Mejia-Ruiz, G., Paternina, M. R. A., Rodriguez, J. R., Zamora, A., Bolivar-Ortiz, G. & Toledo-Santos, C. A Bidirectional isolated charger for electric vehicles in V2G Systems with the capacity to provide ancillary services. in *2020 52nd North American Power Symposium, NAPS 2020* (2021). 10.1109/NAPS50074.2021.9449674

[122] Türkoğlu, A. S. et al. Maximizing EV profit and grid stability through virtual power plant considering V2G. *Energy Rep.* **11**, 3509–3520. 10.1016/j.egyr.2024.03.013 (2024).

[123] Wang, H., Jia, Y., Shi, M., Lai, C. S. & Li, K. A mutually beneficial operation framework for virtual power plants and electric vehicle charging stations. *IEEE Trans. Smart Grid* **14**(6), 4634–4648. 10.1109/TSG.2023.3273856 (2023).

[124] Metwly, M. Y., Hamad, M. S., Abdel-Khalik, A. S. & Eldesouki, H. A review of power management strategies for grid frequency regulation using electric vehicles. in *2021 22nd International Middle East Power Systems Conference (MEPCON)* (IEEE, 2021). 541–547. 10.1109/MEPCON50283.2021.9686203

[125] Heilmann, C. & Friedl, G. Factors influencing the economic success of grid-to-vehicle and vehicle-to-grid applications: A review and meta-analysis. *Renew. Sustain. Energy Rev.*10.1016/j.rser.2021.111115 (2021).

[126] Metwly, M. Y. et al. Power management optimization of electric vehicles for grid frequency regulation: Comparative study. *Alex. Eng. J.*10.1016/j.aej.2022.10.030 (2023).

[127] Tamura, S. A V2G strategy to increase the cost–benefit of primary frequency regulation considering EV battery degradation. *Electr. Eng. Jpn.* **212**(1–4), 11–22. 10.1002/eej.23270 (2020).

[128] Kaur, K., Kumar, N. & Singh, M. Coordinated power control of electric vehicles for grid frequency support: MILP-Based hierarchical control design. *iEEE Trans. Smart Grid* **10**(3), 3364–3373. 10.1109/TSG.2018.2825322 (2019).

[129] Amamra, S.-A. & Marco, J. Vehicle-to-grid aggregator to support power grid and reduce electric vehicle charging cost. *IEEE Access* **7**, 178528–178538. 10.1109/ACCESS.2019.2958664 (2019).

[130] Wang, M. et al. State space model of aggregated electric vehicles for frequency regulation. *IEEE Trans. Smart Grid* **11**(2), 981–994. 10.1109/TSG.2019.2929052 (2020).

[131] Cui, Y., Hu, Z. & Luo, H. Optimal day-ahead charging and frequency reserve scheduling of electric vehicles considering the regulation signal uncertainty. *IEEE Trans. Ind. Appl.* **56**(5), 5824–5835. 10.1109/TIA.2020.2976839 (2020).

[132] Cai, S. & Matsuhashi, R. Model predictive control for EV aggregators participating in system frequency regulation market. *IEEE Access* **9**, 80763–80771. 10.1109/ACCESS.2021.3085345 (2021).

[133] Wang, X., He, Z. Y. & Yang, J. W. Unified strategy for electric vehicles participate in voltage and frequency regulation with active power in city grid. *IET Generat. Transm. Distrib.* **13**(15), 3281–3291. 10.1049/iet-gtd.2018.7016 (2019).

[134] ur Rehman, U. A robust vehicle to grid aggregation framework for electric vehicles charging cost minimization and for smart grid regulation. *Int. J. Electr. Power Energy Syst.*10.1016/j.ijepes.2022.108090 (2022).

[135] Yumiki, S. et al. Autonomous vehicle-to-grid design for provision of frequency control ancillary service and distribution voltage regulation sustainable energy. *Grids Netw.*10.1016/j.segan.2022.100664 (2022).

[136] Ma, K. et al. Voltage Regulation with electric taxi based on dynamic game strategy. *IEEE Trans. Veh. Technol.* **71**(3), 2413–2426. 10.1109/TVT.2022.3141954 (2022).

[137] Aziz, N., Shah, M. A. & Mehmood, M. U. Vehicle to grid (V2G) for peak shaving: New trend, benefits, and issues. *Int. J. Comput. Commun. Netw.* **1**(2), 27–37 (2019).

[138] Ding, Y., Li, X. & Jian, S. Modeling the impact of vehicle-to-grid discharge technology on transport and power systems. *Transp. Res. D Transp. Environ.*10.1016/j.trd.2022.103220 (2022).

[139] Ehsani, M., Falahi, M. & Lotfifard, S. Vehicle to grid services: Potential and applications. *Energies (Basel)*10.3390/en5104076 (2012).

[140] Imani, M. H., Yousefpour, K., Ghadi, M. J. & Andani, M. T. Simultaneous presence of wind farm and V2G in security constrained unit commitment problem considering uncertainty of wind generation. in *2018 IEEE Texas Power and Energy Conference, TPEC 2018* (2018). 10.1109/TPEC.2018.8312082

[141] Deb, S., Goswami, A. K., Chetri, R. L. & Roy, R. Congestion management considering plug-in electric vehicle charging coordination in distribution system. in *2021 International Conference on Nascent Technologies in Engineering, ICNET 2021—Proceedings* (2021). 10.1109/ICNTE51185.2021.9487681

[142] López, M. A., Martín, S., Aguado, J. A. & De La Torre, S. V2G strategies for congestion management in microgrids with high penetration of electric vehicles. *Electr. Power Syst. Res.*10.1016/j.epsr.2013.06.005 (2013).

[143] Deb, S. et al. Charging Coordination of plug-in electric vehicle for congestion management in distribution system integrated with renewable energy sources. *IEEE Trans. Ind. Appl.*10.1109/TIA.2020.3010897 (2020).

[144] Golden, R. & Paulos, B. Curtailment of renewable energy in california and beyond. *Electr. J.*10.1016/j.tej.2015.06.008 (2015).

[145] Bird, L., Cochran, J. & Wang, X. *Wind and Solar Energy Curtailment: Experience and Practices in the United States* (National Renewable Energy Laboratory (NREL), 2014).

[146] Sovacool, B. K., Noel, L., Axsen, J. & Kempton, W. The neglected social dimensions to a vehicle-to-grid (V2G) transition: A critical and systematic review. *Environ. Res. Lett.*10.1088/1748-9326/aa9c6d (2018).

[147] Chauhan, A. & Saini, R. P. A review on integrated renewable energy system based power generation for stand-alone applications: Configurations, storage options, sizing methodologies and control. *Renew. Sustain. Energy Rev.* **38**, 99–120. 10.1016/j.rser.2014.05.079 (2014).

[148] Zhou, C., Qian, K., Allan, M. & Zhou, W. Modeling of the cost of EV battery wear due to V2G application in power systems. *IEEE Trans. Energy Convers.* **26**(4), 1041–1050. 10.1109/TEC.2011.2159977 (2011).

[149] Darcovich, K., Laurent, T. & Ribberink, H. Improved prospects for V2X with longer range 2nd generation electric vehicles. *eTransportation* **6**, 100085. 10.1016/j.etran.2020.100085 (2020).

[150] Vatanparvar, K. & Al Faruque, M. A. Battery lifetime-aware automotive climate control for electric vehicles. in *Proceedings of the 52nd Annual Design Automation Conference* 1–6 (ACM, 2015). 10.1145/2744769.2744804

[151] Blanes, J. M., Gutiérrez, R., Garrigós, A., Lizán, J. L. & Cuadrado, J. M. Electric vehicle battery life extension using ultracapacitors and an FPGA controlled interleaved buck-boost converter. *IEEE Trans. Power Electron.* **28**(12), 5940–5948. 10.1109/TPEL.2013.2255316 (2013).

[152] Zakzouk, N. E. & Lotfi, R. A. Power flow control of a hybrid battery/supercapacitor standalone PV system under irradiance and load variations. in *2020 10th International Conference on Power and Energy Systems (ICPES)* 469–474 (IEEE, 2020). 10.1109/ICPES51309.2020.9349655

[153] Tasnim, M. N. et al. A critical review on contemporary power electronics interface topologies to vehicle-to-grid technology: Prospects, challenges, and directions. *IET Power Electron.* **17**(1), 157–181. 10.1049/pel2.12618 (2024).

[154] Mohammed, A., Saif, O., Abo-Adma, M., Fahmy, A. & Elazab, R. Strategies and sustainability in fast charging station deployment for electric vehicles. *Sci. Rep.*10.1038/s41598-023-50825-7 (2024). 38168937 10.1038/s41598-023-50825-7 PMC10762045

[155] Van Der Kam, M. & Bekkers, R. Mobility in the smart grid: Roaming protocols for EV charging. *IEEE Trans. Smart Grid* **14**(1), 810–822. 10.1109/TSG.2022.3202608 (2023).

[156] I. Energy Agency, Grid integration of electric vehicles: A manual for policy makers. www.iea.org

[157] Günzel-Jensen, F. & Rask, M. Combating climate change through collaborations? Lessons learnt from one of the biggest failures in environmental entrepreneurship. *J. Clean. Prod.* **278**, 123941. 10.1016/j.jclepro.2020.123941 (2021).

[158] Costa, E., Wells, P., Wang, L. & Costa, G. The electric vehicle and renewable energy: Changes in boundary conditions that enhance business model innovations. *J Clean Prod* **333**, 130034. 10.1016/j.jclepro.2021.130034 (2022).

[159] Krishna, G. Understanding and identifying barriers to electric vehicle adoption through thematic analysis. *Transp. Res. Interdiscip. Perspect.* **10**, 100364. 10.1016/j.trip.2021.100364 (2021).

[160] Chen, Z., Carrel, A. L., Gore, C. & Shi, W. Environmental and economic impact of electric vehicle adoption in the U.S. *Environ. Res. Lett.*10.1088/1748-9326/abe2d0 (2021).35330988

[161] Figenbaum, E. Retrospective Total cost of ownership analysis of battery electric vehicles in Norway. *Transp. Res. D Transp. Environ.*10.1016/j.trd.2022.103246 (2022).

[162] Beatty, T. K. M. & Shimshack, J. P. School buses, diesel emissions, and respiratory health. *J. Health Econ.* **30**(5), 987–999. 10.1016/j.jhealeco.2011.05.017 (2011). 21741102 10.1016/j.jhealeco.2011.05.017

[163] Lebeau, K., Lebeau, P., Macharis, C. & Van Mierlo, J. How expensive are electric vehicles? A total cost of ownership analysis. in *2013 World Electric Vehicle Symposium and Exhibition (EVS27)* 1–12 (IEEE, 2013). 10.1109/EVS.2013.6914972

[164] Dall-Orsoletta, A., Ferreira, P. & Gilson Dranka, G. Low-carbon technologies and just energy transition: Prospects for electric vehicles. *Energy Convers. Manag.: X*10.1016/j.ecmx.2022.100271 (2022).

[165] Chandra Tripathi, G. A Literature review of electric vehicle to grid technology. http://www.webology.org

[166] Lin, B. & Wu, W. The impact of electric vehicle penetration: A recursive dynamic CGE analysis of China. *Energy Econ.*10.1016/j.eneco.2020.105086 (2021).

[167] Asadi, S. et al. Factors impacting consumers’ intention toward adoption of electric vehicles in Malaysia. *J. Clean. Prod.*10.1016/j.jclepro.2020.124474 (2021).

[168] Singh, V., Singh, T., Higueras-Castillo, E. & Liebana-Cabanillas, F. J. *Sustainable Road Transportation Adoption Research: A Meta and Weight Analysis, and Moderation Analysis* (Elsevier Ltd, 2023). 10.1016/j.jclepro.2023.136276.

[169] Chen, M. et al. Recycling End-of-life electric vehicle lithium-ion batteries. *Joule* **3**(11), 2622–2646. 10.1016/j.joule.2019.09.014 (2019).

[170] Palaniswamy, S., SandhyaDevi, R. S., Saravanan, M. & Anand, M. *Social Economic and Environmental Impact of Electric Vehicles in India* (University of the Philippines at Los Banos, 2022). 10.47125/jesam/2022_1/08.

[171] Zaino, R., Ahmed, V., Alhammadi, A. M. & Alghoush, M. Electric vehicle adoption: A comprehensive systematic review of technological, environmental, organizational and policy impacts. *World Electr. Veh. J.* **15**(8), 375. 10.3390/wevj15080375 (2024).

[172] Alanazi, F. Electric vehicles: Benefits, challenges, and potential solutions for widespread adaptation. *Appl. Sci.* **13**(10), 6016. 10.3390/app13106016 (2023).

